# EGF-Induced miR-223 Modulates Goat Mammary Epithelial Cell Apoptosis and Inflammation via *ISG15*

**DOI:** 10.3389/fcell.2021.660933

**Published:** 2021-06-30

**Authors:** Yue Zhang, Qiong Wu, Guanglin Niu, Jidan Liu, Fangjun Cao, Xiaopeng An, Binyun Cao

**Affiliations:** ^1^College of Animal Science and Technology, Northwest A&F University, Xianyang, China; ^2^TUM School of Life Sciences, Technical University of Munich, Freising, Germany; ^3^Medical College, Qinghai University, Xining, China

**Keywords:** EGF, miR-223, *ISG15*, apoptosis, inflammation, mammary gland

## Abstract

The health of mammary gland is essential for lactation. Epidermal growth factor (EGF) is reported to play an important role in lactation initiation and miR-223 is a conserved microRNA in anti-inflammation. In this study, EGF was found to induce a higher expression of miR-223 in goat mammary epithelial cell (gMEC). The downstream genes of miR-223 were screened by RNA sequencing, including *Interferon-stimulated gene product 15* (*ISG15*), a pivotal immune responder, which was detected to be downregulated by EGF and miR-223. Due to the correlation between inflammation and apoptosis, the gMEC apoptosis modulated by EGF, miR-223, and *ISG15* was investigated, and the protein expressions of Bcl-2/Bax, Caspase 3 and p53 were examined to evaluate the apoptosis of gMEC. The protein expressions of p-STAT3/STAT3, PR, FOXC1, and HOXA10, which had been shown to be related to inflammation, were detected to assess the inflammation of gMEC. This study provided a regulation axis, EGF/miR-223/*ISG15*, and illustrated its regulation to gMEC apoptosis and inflammation.

## Introduction

Mammary gland is a complex tissue undergoing constant remodeling. It is characterized by highly branched tubular structures with luminal cells surrounded by myoepithelial cells during puberty. The mammary epithelium expands massively during pregnancy to form alveolar structures, which would differentiate into secretary units in late pregnancy to achieve lactation (Watson, 2006). At the onset of lactation, the expression of epidermal growth factor (EGF) surges compared to late pregnancy (Fu et al., 2015), indicating the importance of EGF in the lactation process.

MicroRNAs (miRNAs) are a class of endogenous non-coding RNAs about 22 nucleotides in length that function by repressing the expression of their target genes after transcription (Jonas and Izaurralde, 2015). MiRNAs play critical roles in animal development and physiology (Ambros, 2004), especially in mammary gland (Ji et al., 2019; Xuan et al., 2020; Zhang et al., 2020b,2021b). In this study, we found the expression of miR-223 could be induced by EGF in goat mammary epithelial cells (gMEC). Through RNA-sequencing and analysis of downstream differentially expressed genes (DEGs) of miR-223, the functions and pathways that miR-223 involved in were enriched. In the light of the enrichment, miR-223 was found to be highly associated with inflammation, which was consistent with previous studies (Haneklaus et al., 2013; Jeffries et al., 2019). *Interferon-stimulated gene product 15* (*ISG15*) is one of the DEGs with the highest expression in negative control (NC) group and the second greatest fold-change between miR-223 and NC groups. As the first identified ubiquitin-like protein, ISG15 is a pivotal component of host responses to microbial infection that actively involves in inflammation (Zhang et al., 2015; Dzimianski et al., 2019), which is correlated with apoptosis (Vasilikos et al., 2017).

Here we explored the relationship among EGF, miR-223, and *ISG15*, and their involvement in the apoptosis and inflammation of gMEC. The gMEC apoptosis regulated by EGF, miR-223, and *ISG15* was measured by flow cytometry and evaluated by the expression of classical apoptosis-related proteins (Zamzami and Kroemer, 2005; Ola et al., 2011) Bcl-2/Bax, p53, and Caspase 3. Then inflammation-related protein expressions (p-STAT3, PR, FOXC1, and HOXA10) were examined to evaluate the involvement of EGF, miR-223 and *ISG15* in gMEC inflammation. Signal transducers and activators of transcription 3 (STAT3) is a cellular signal transcription factor involved in many cellular activities, and phosphorylated (p-) STAT3 on Ser727 is a crucial intermediary for inflammatory cytokine production (Hu et al., 2019a; Balic et al., 2020). Progesterone (P4) is one of the main hormones involved in mammary gland remodeling during lactation and implicated in host immune regulation (Xu et al., 2017). Previous studies revealed that progesterone receptor (PR) could protect cells from inflammation (Park et al., 2020). Forkhead box C1 (FOXC1) is reported to attenuate the inflammation in chronic obstructive pulmonary disease (Xia et al., 2019), and homeobox 10 (*HOXA10*) is demonstrated to contribute to the innate immune response and suppress inflammation (Wang et al., 2015). The pathways involved in the regulation of apoptosis and inflammation by EGF, miR-223 and *ISG15* were assessed by detecting the expression of these proteins.

## Materials and Methods

### Animals

One cubic centimeter of mammary gland tissue was taken with a scalpel from 3-year-old Guanzhong dairy goats in peak lactation period after anesthetizing and stored in PBS with penicillin/streptomycin (100 U/mL, Harbin Pharmaceutical Group, China). The wound was sewed and sterilized immediately, and the goats recovered after a week. The mammary gland tissue was used to isolate gMECs as reported (Wang et al., 2010). Specifically, the tissue was cut into pieces around 1 mm^3^ and seeded in cell culture plates, and then gMECs would grow adherently. One week later, gMECs were obtained and purified. All the procedures conformed to the guidelines of the Animal Care and Use Committee of the Northwest A&F University (ethic code: #0726/2018).

### Vector Construction

The full coding sequence of *ISG15* was amplified with forward primer: 5′-cccaagcttgccaccATGGGCGGGGACCTGAAG-3′ and reverse primer: 5′-ccgctcgag CTACCCACCCCGCAG-3′ using 2 × HiFiTaq PCR StarMix with Loading Dye (Genstar, Beijing, China), and inserted into pcDNA3.1 (+) vector between the restriction sites Hind III and Xho I to achieve the overexpression of *ISG15*. The segment of *ISG15* containing the seed site of miR-223 was amplified with forward primer: 5′-ccgctcgag ACCTTGACAGCAGGGAAGTG-3′ and reverse primer: 5′-ataagaatgcggccgcCAGAATTGGTCCGCTTGCAC-3′ using 2 × HiFiTaq PCR StarMix with Loading Dye (Genstar, Beijing, China), and inserted to a dual-luciferase reporter vector, psiCHECK2, between the restriction sites Xho I and Not I, for dual-luciferase reporter assay.

### Cell Culture

Primary gMECs were cultured in DMEM/F12 medium (Hyclone, United States) with 10% fetal bovine serum and incubated at 37°C with 5% CO_2_ in a humid environment. The sRNAs and vectors were transfected into gMECs using Lipofectamine 2000 reagent (Invitrogen, United States), and the transfection was conducted when gMEC density reached about 80%. One hundred pmol sRNA (or 4 μg vector) and 5 μl Lipofectamine 2000 were diluted into 250 μl Opti-MEM (Gibco, United States), respectively, and sRNA or vector was gently mixed into Lipofectamine 2000 and incubated for 20 min at room temperature. The mixture was applied to gMECs in six-well plates. Four hours later, the medium was changed to the fresh medium. Forty-eight hours post-transfection, the gMECs were harvested. The sequences of sRNAs are given below. Negative control (NC): 5′-UUCUCCGAACGUGUCACGUTT-3′; inhibitor NC: 5′-CAGUACUUUUGUGUAGUACAA-3′; miR-223 mimic: 5′-UGUCAGUUUGUCAAAUACCCCA-3′; miR-223 inhibitor: 5′-UGGGGUAUUUGACAAACUGACA-3′; si*ISG15*: 5′-GCGUGUGCAAGCGGACCAATT-3′. MiR-223 mimic was applied for the overexpression of miR-223, miR-223 inhibitor was for the inhibition of the expression of miR-223, and si*ISG15* was for the knockdown of the expression of *ISG15*.

### Dual-Luciferase Reporter Assay

The plasmid of psiCHECK2-*ISG15* vector was co-transfected with miR-223 or negative control (NC), respectively, into gMEC. After 48 h, cells were lysed with passive lysis buffer, and the Firefly luciferase (*hluc*^+^) activity and Renilla luciferase (*hRluc*) activity were measured by a Dual-Luciferase Reporter Assay System (Promega, Madison, WI, United States) according to the manufacturers’ instruction. The ratio of *hRluc* and *hluc*^+^ was calculated to evaluate the relative luciferase activity.

### RNA Isolation and Analysis

The gMECs were washed mildly by PBS and lysed using TRIzol Reagent (Invitrogen, United States) for RNA isolation. The total RNA was quality-controlled by Agilent bioanalyzer 2100 and applied for cDNA synthesis of mRNA by the PrimeScript RT Reagent Kit with gDNA Eraser (Takara, Japan). The cDNA of miRNA was acquired using the miRcute Plus miRNA First-Strand cDNA Kit (Tiangen, Beijing, China). RT-qPCR was conducted using SYBR Green qPCR Master Mix (Takara, Japan) with following primers: 5′-TTTGCAGCAGGTCAGAGAGA-3′ (*MX1* forward); 5′-GTACG CCATCAGGTGTTGAA-3′ (*MX1* reverse); 5′-CCAATCAGATC CCGTTCATC-3′ (*MX2* forward); 5′-CCTGAAGCAGCCAGG AATAG-3′ (*MX2* reverse); 5′-ACCTTGACAGCAGGGAAGTG-3′ (*ISG15* forward); 5′-GTCGTTCCTCACCAGGATGT-3′ (*ISG 15* reverse); 5′-GTGCATGGTGTTTCAGATGC-3′ (*DDX58* for ward); 5′-TCCGTGCATCCTCACTGATA-3′ (*DDX58* reverse); 5′-ACTCCCACCAGCGTCAATTA-3′ (*RSAD2* forward); 5′-AG ACCTCTCTTGGCCTCCTC-3′ (*RSAD2* reverse); 5′-CCAATT ACACCCAAGCATCC-3′ (*IFI44L* forward); 5′-GTTTGAGCTT CGCCATCATT-3′ (*IFI44L* reverse); 5′-CTGGCCATCGCAA TGTACTA-3′ (*IFIT3* forward); 5′-AGGGCCAGGAGAACTTTG AT-3′ (*IFIT3* reverse); 5′-GTGGCCAAGGACAACAAGAT-3′ (*F HL1* forward); 5′-GTGCCCTTGTATTCCACGTT-3′ (*FHL1* rev erse); 5′-GATCTGGCACCACACCTTCT-3′ (β-*actin* forward); 5′-GGGTCATCTTCTCACGGTTG-3′ (β-*actin* reverse); 5′-GCG TGTCAGTTTGTCAAATACCCCA-3′ (miR-223 forward); the reverse primer of miRNA was provided by Tiangen (Beijing, China). The Poly (A) mRNA Magnetic Isolation Module (NEBNext, United States) was used to capture mRNA from total RNA, and Ultra RNA Library Prep Kit for Illumina (NEBNext, United States) was applied for the library construction. Then the library was purified and quality-controlled for RNA-sequencing, which was performed by Illumina HiSeq 2500. The obtained raw reads were filtered for clean reads of high quality, which were mapped to the reference genome database^1^. The differentially expressed genes were screened, and GO and KEGG enrichments were conducted.

### Flow Cytometry

The gMECs were digested by pancreatin free of EDTA and washed by PBS subsequently. The cells were centrifuged at 1,000 rpm for 5 min and resuspended with 1 × Binding Buffer (7Sea Biotech, Shanghai, China). The apoptosis was examined using the Annexin V-FITC/PI apoptosis kit (7Sea Biotech, Shanghai, China). Within 1 h after the staining, the apoptotic rate was measured by a flow cytometer (BD, United States). The apoptosis results were shown in a four-quadrant diagram: dots in lower left quadrant represented normal cells; dots in upper left quadrant represented necrotic cells; dots in lower right quadrant represented late apoptotic cells; dots in upper right represented early apoptotic cells. The total apoptotic rate was calculated to evaluate the apoptosis of gMEC.

### Western Blot

The total protein of gMEC was harvested by using RIPA lysis buffer with protease inhibitor and phosphatase inhibitor (Thermo Fisher Scientific, United States). The concentration of the proteins was measured by a BCA protein assay kit (Solarbio, Beijing, China). Equal amount of protein was loaded to a 12% SDS-PAGE gel. Primary antibodies used were as follows: anti-Bcl-2 (BBI, D260117, Shanghai, China), anti-Bax (BBI, D220073, Shanghai, China), anti-p53 (Beyotime, AF7671, Shanghai, China), anti-caspase 3 (Cell Signaling Technology, 9665, United States), anti-p-STAT3 (BBI, Phospho-Ser727, D155018, Shanghai, China), anti-STAT3 (BBI, D320083, Shanghai, China), anti-PR (Beyotime, AF7737, Shanghai, China), anti-FOXC1 (BBI, D160347, Shanghai, China), anti-HOXA10 (BBI, D163629, Shanghai, China), anti-β-actin (Beyotime, AA128, Shanghai, China). Horseradish peroxidase conjugated anti-mouse (Beyotime, A0216, Shanghai, China) and anti-rabbit (Beyotime, A0208, Shanghai, China) antibodies were applied as secondary antibodies.

### Statistics

The quality of sequencing data was evaluated using FastQC v0.10.1, and raw reads were filtered by Cutadapt 1.9.1. Short read alignment was performed by Hisat v2.0.14. DEGs were conditioned on a more than twofold difference and FDR < or = 0.05. GO function and KEGG pathway annotation analyses were based on the GO database^2^ and the KEGG database^3^, respectively. All the experiments were repeated at least three times independently. Statistical significance was calculated via Student’s *t*-test and one-way ANOVA (SPSS 22.0; SPSS Inc). Significance: ^∗^*p* < 0.05, ^∗∗^*p* < 0.01.

## Results

### MiR-223 Induced by EGF Inhibits gMEC Apoptosis

**FIGURE 1:**
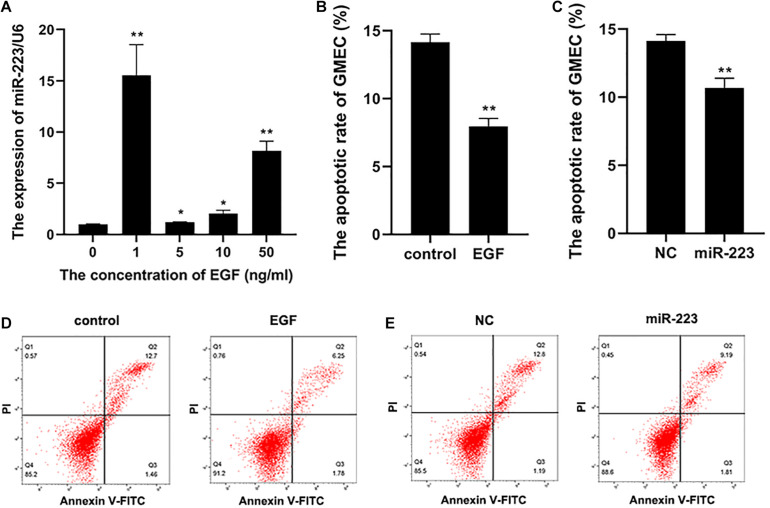
The apoptosis of gMEC regulated by EGF and miR-223. **(A)** A higher expression of miR-223 was induced by EGF. The gMECs were incubated for 48 h with EGF at concentrations of 0, 1, 5, 10, and 50 ng/ml, respectively, and the expression of miR-223 was then detected, which revealed that the expression of miR-223 was the highest in gMEC incubated with 1 ng/ml of EGF. **(B,D)** The regulation of gMEC apoptosis by EGF. The cell cultivators with or without EGF at 1 ng/ml were applied to incubate gMEC for 48 h, and a reduction of apoptotic rate in gMEC was found in EGF group. **(C,E)** The regulation of gMEC apoptosis by miR-223. MiR-223 and NC were transfected into gMEC, respectively, and 48 h post-transfection gMEC apoptotic rate was found reduced in miR-223 group. *p < 0.05; **p < 0.01.

Epidermal growth factor was diluted into cell cultivator at concentrations of 0, 1, 5, 10, and 50 ng/ml to incubate gMEC for 48 h. Concentration of EGF at 0 ng/ml was set as the control group. The result revealed that 1 ng/ml of EGF was the most effective to induce a higher expression of miR-223 (Figure 1A). Therefore, 1 ng/ml of EGF was applied to the following experiments. The apoptotic rate of gMECs incubated with 1 ng/ml of EGF was measured, which illustrated an inhibitory effect of EGF on gMEC apoptosis (Figures 1B,D). To explore the effect of miR-223 on gMEC apoptosis, miR-223 and negative control (NC) was transfected into gMEC, and gMEC apoptotic rate was measured after 48 h. The result revealed that miR-223 suppressed the apoptosis of gMEC (Figures 1C,E).

### Screening of miR-223 Downstream Genes

**FIGURE 2:**
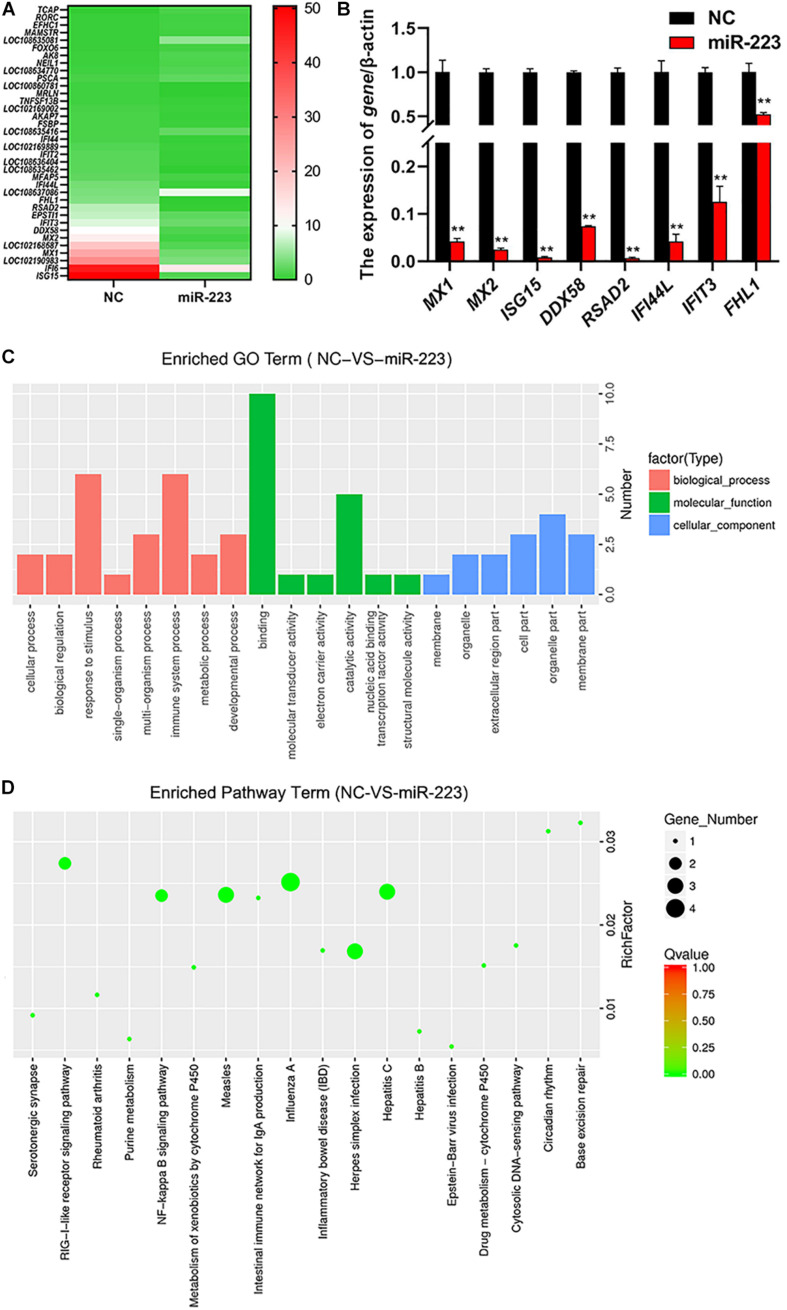
The analysis of differentially expressed genes induced by miR-223. **(A)** The downstream genes regulated by miR-223. MiR-223 and NC were transfected into gMECs, respectively, and gMECs were harvested after 48 h for RNA-seq to screen differentially expressed genes of miR-223, which were shown in the heatmap. **(B)** The accuracy test of RNA-seq by RT-qPCR. Eight differentially expressed genes were randomly chosen to ensure the accuracy of the RNA sequencing by RT-qPCR, and the result showed that the result of RNA-seq was reliable. **(C,D)** GO analysis and KEGG pathway analysis of differentially expressed genes of miR-223 were conducted to predict the function of miR-223. It showed that the differentially expressed genes involved in 20 GO terms **(C)**, and 18 KEGG pathways **(D)**. **p < 0.01.

MiR-223 mimic and inhibitor were transfected into gMEC, respectively, and NC and inhibitor NC were transfected, respectively, as control. Total RNA of gMEC was isolated 48 h post-transfection. The efficiency of miR-223 mimics and miR-223 inhibitor was measured (Supplementary Figure 1). Groups miR-223 mimic and NC were used for RNA-seq (Sequence Read Archive: SRR13638390) to screen differentially expressed genes (DEGs). Thirty-six DEGs were found, including 24 downregulated genes and 12 upregulated genes. The heatmap of DEGs is shown in Figure 2A. To ensure the accuracy of RNA-sequencing, eight of the DEGs were randomly picked to perform RT-qPCR (Figure 2B), which ensured the reliability of the result of RNA-sequencing and demonstrated the down-regulation of *ISG15* by miR-223. GO and KEGG enrichments of DEGs were conducted to predict the function of miR-223. GO enrichment showed that miR-223 might involve in 20 GO terms (Figure 2C), such as binding (GO:0003723), catalytic activity (GO:0003824), response to stimulus (GO:0050896) and immune system process (GO:0002376), and 18 KEGG pathways that miR-223 could participate in were found (Figure 2D), including RIG-I like receptor signaling pathway (ko04622).

### MiR-223 Induces gMEC Apoptosis via *ISG15*

**FIGURE 3:**
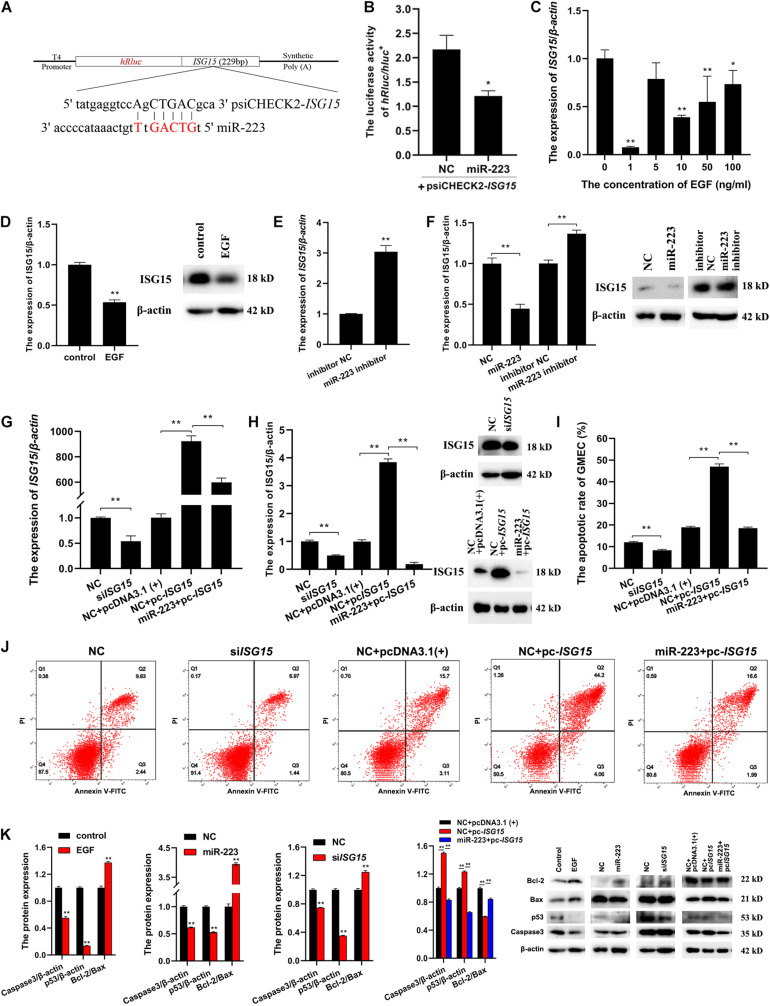
A further investigation of the relationships among EGF, miR-223 and *ISG15*, and their regulation to apoptosis. **(A)** The information of psiCHECK2-*ISG15* vector for dual luciferase reporter assay. The sequence of *ISG15* containing seed site of miR-223 was amplified and inserted into psiCHECK2 vector. **(B)** The relative luciferase activity regulated by miR-223. PsiCHECK2-*ISG15* vector was co-transfected with miR-223 or NC, respectively, and 48 h later, a decreased relative luciferase activity was detected in miR-223 group. **(C,D)** The effect of EGF on the expression of *ISG15* in gMEC. EGF at 0, 1, 5, 10, and 50 ng/ml was applied to incubate gMEC, and the expression of *ISG15* was examined 48 h later. The group with 1 ng/ml of EGF witnessed the lowest expression of *ISG15* **(C)**, and the protein expression of ISG15 was also decreased by 1 ng/ml of EGF **(D)**. **(E,F)** The regulation of *ISG15* by miR-223. The expression of *ISG15* regulated by miR-223 had been evaluated in the accuracy test of RNA-seq, and the inhibitor of miR-223 was applied to enhance the conclusion that miR-223 downregulated the expression of *ISG15* **(E)**. The measurement of protein expression of ISG15 modulated by miR-223 illustrated a reduction of ISG15 induced by miR-223 **(F)**. **(G,H)** The efficiency of si*ISG15* and pc-*ISG15*. To explore the function of *ISG15* in gMEC, the efficiency of si*ISG15* and pc-*ISG15* was detected by RT-qPCR **(G)** and Western Blot **(H)**, which showed the effectiveness of si*ISG15* and pc-*ISG15*. **(I,J)** The apoptotic rate of gMEC regulated by *ISG15*. Flow cytometry was used to detect gMEC apoptosis, and it showed that si*ISG15* suppressed gMEC apoptosis and pc-*ISG15* enhanced gMEC apoptosis, while miR-223 impaired the promotion of apoptosis by pc-*ISG15*. **(K)** The protein expression of apoptosis-related biomarkers (Bcl-2/Bax, p53, caspase 3). EGF, miR-223, and si*ISG15* increased the expression of Bcl-2/Bax and reduced the expression of p53 and Caspase 3, and pc-*ISG15* reduced the expression of Bcl-2/Bax and elevated the expression of p53 and Caspase 3, while miR-223 weakened its regulation. *p < 0.05; **p < 0.01.

To investigate whether *ISG15* could be targeted by miR-223, the sequence of *ISG15* containing the seed site of miR-223 was amplified and inserted into psiCHECK2 (Figure 3A). The psiCHECK2-*ISG15* vector was co-transfected with miR-223 and NC, respectively, into gMEC. The luciferase activities were detected after 48 h, and it showed a decreased relative luciferase activity in the miR-223 group (Figure 3B), which means *ISG15* was targeted by miR-223. It is shown in Figure 3C that EGF reduced the expression of *ISG15*, especially at 1 ng/ml, and EGF at 1 ng/ml decreased the protein expression of ISG15 as well (Figure 3D). The miR-223 inhibitor and inhibitor NC were transfected into gMEC and the expression of *ISG15* was measured after 48 h. The increase of *ISG15* expression in miR-223 inhibitor group reassured the down-regulation of *ISG15* by miR-223 (Figure 3E). It is shown in Figure 3F that miR-223 could restrain the protein expression of ISG15, and when miR-223 was inhibited, the protein expression of ISG15 was increased.

To explore the effect of *ISG15* on gMEC, the efficiency of *ISG15* siRNA (si*ISG15*) and pcDNA3.1-*ISG15* vector (pc-*ISG15*) was ensured by RT-qPCR and Western Blot. Meanwhile, the efficiency of pc-*ISG15* decreased when co-transfected with miR-223, which further confirmed the inhibitory effect of miR-223 on *ISG15* expression (Figures 3G,H). The apoptotic rate of gMEC was measured by flow cytometry to investigate the regulation of gMEC apoptosis by *ISG15*. The results showed reduced apoptotic rates in si*ISG15* group and increased apoptotic rates in pc-*ISG15* group. The apoptosis of gMEC induced by pc-*ISG15* was alleviated by co-transfection with miR-223 (Figures 3I,J). The expression of apoptosis-related proteins, like p53, Caspase 3, and Bcl-2/Bax regulated by EGF/miR-223/*ISG15* axis were detected to further explore the regulation of gMEC apoptosis. The results showed that the expressions of p53 and Caspase 3 were decreased, and the expression of Bcl-2/Bax was increased by EGF, miR-223 and si*ISG15*. The overexpression of *ISG15* enhanced the expressions of p53 and Caspase 3 and reduced the expression of Bcl-2/Bax, while miR-223 mitigated the regulation of apoptosis-related proteins by pc-*ISG15* (Figure 3K).

### The Regulation of EGF/miR-223/*ISG15* Axis to Inflammation

**FIGURE 4:**
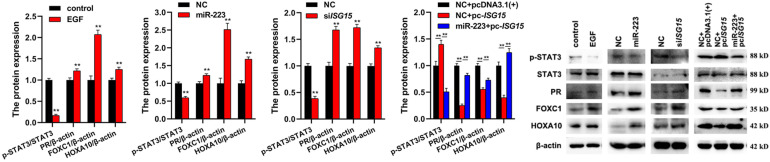
Effects of EGF, miR-223, and *ISG15* on gMEC inflammation. The regulation of inflammation-related protein expressions (p-STAT3, PR, FOXC1, and HOXA10) by EGF, miR-223, and *ISG15* was detected, and it showed that EGF, miR-223, and si*ISG15* significantly decreased the expression of p-STAT3 and elevated the expressions of PR, FOXC1, and HOXA10 in gMEC. Pc-*ISG15* promoted the expression of p-STAT3 and reduced the expressions of PR, FOXC1, and HOXA10 in gMEC, while miR-223 weakened its regulation. EGF has been reported to surge at the onset of lactation, suggesting its importance in the lactation process. In this study, we found that the expression of miR-223, a conserved anti-inflammatory miRNA, could be induced by EGF. The downstream differentially expressed genes of miR-223 were screened by RNA-seq, and abundant interferon stimulated genes were found to be regulated by miR-223, including *ISG15*, a pivotal immune responder. Therefore, we explored the role of EGF, miR-223, and *ISG15* in goat mammary epithelial cell apoptosis and inflammation to elaborate how mammary gland could be protected from mastitis during lactation. **p < 0.01.

To investigate the involvement of EGF, miR-223 and *ISG15* in inflammation, the expressions of inflammation-related proteins (p-STAT3, PR, FOXC1, and HOXA10) were detected by Western Blot. The results showed that EGF, miR-223, and si*ISG15* significantly decreased the expression of p-STAT3 and elevated the expressions of PR, FOXC1, and HOXA10 in gMEC (Figure 4). When the *ISG15* was overexpressed, the expression of p-STAT3 increased and the expressions of PR, FOXC1 and HOXA10 reduced, and miR-223 could impair the effect of *ISG15* overexpression on the protein expressions in gMEC (Figure 4).

## Discussion

Our data showed that EGF could significantly elevate the expression of miR-223 in gMEC, especially at 1 ng/ml. Downstream differentially expressed genes (DEGs) between miR-223 and negative control (NC) groups were screened by RNA-seq. The innate immune response mediated by interferon is a robust first line of defense against pathogens (Schneider et al., 2014). Interferon signaling is important in immune response and inflammation (Chen et al., 2017; Zhang et al., 2017). The abundance of interferon-stimulated genes in the downregulated DEG list, such as *interferon-stimulated gene product 15* (*ISG15*), *IFI6*, *MX1*, *MX2*, *DDX58*, *IFIT3*, *RSAD2*, *IFI44L*, *IFIT2*, and *IFI44*, indicates that miR-223 was likely to participate in immunity and inflammation. *ISG15* was one of the DEGs with the highest expression in the NC group and was greatly downregulated by miR-223. The downregulation of *ISG15* by miR-223 was confirmed by RT-qPCR and Western Blot. The targeting regulation of miR-223 to *ISG15* was illustrated by a dual luciferase reporter assay. It was demonstrated that the expression of *ISG15* was also reduced by epidermal growth factor (EGF), which suggested the expression of *ISG15* could be reduced by EGF-induced miR-223. The apoptotic rate was measured by flow cytometry and the expressions of anti-apoptotic Bcl-2 (Bruckheimer et al., 1998), and pro-apoptotic caspase 3 (Choudhary et al., 2015) and p53 (Aubrey et al., 2018; Zhang et al., 2020a, 2021a; Niu et al., 2021a,b) were detected to evaluate the apoptosis of gMEC. The inflammation of gMEC was estimated by inflammation-related proteins. It showed that EGF and miR-223 reduced STAT3 phosphorylation levels at Ser727 and promoted the expression of PR, FOXC1, and HOXA10 by downregulating *ISG15*. The results indicated that inhibition of apoptosis and inflammation in gMECs by EGF can be achieved by increasing the expression of miR-223 that can downregulate *ISG15*.

Epidermal growth factor is a 53-amino acid polypeptide mitogen that transduces its signal to EGF receptor (EGFR). The surge of EGF expression at the onset of lactation indicates the importance of EGF in the lactation process (Hardy et al., 2010; Fu et al., 2015). However, studies show that EGF is likely to induce tumor cell invasion in breast cancer (Klijn et al., 1992; Chrysogelos and Dickson, 1994; Hardy et al., 2010), where the overexpression of EGFR is connected with the loss of estrogen receptor and poor prognosis (Chrysogelos and Dickson, 1994). The interesting thing is that EGFR expresses more frequently in normal mammary gland than in malignant tissue (Chrysogelos and Dickson, 1994). The role of EGFR might be different between normal breast and breast cancer. MiR-223 is a guard to prevent relapse of breast cancer (Fabris et al., 2016; Citron et al., 2020). In this study, the expression of miR-223 was raised about 15-fold by EGF, which might be a protection in lactating mammary tissues, and *ISG15*, a critical gene in inflammation (Hu et al., 2019b), was targeted and down-regulated by miR-223. The regulation of gMEC apoptosis and inflammation-related genes by EGF/miR-223/*ISG15* indicated that EGF-induced miR-223 could prevent gMEC apoptosis and inflammation through targeting and down-regulating *ISG15*. In conclusion, our study provided a possible pathway for EGF to protect mammary gland during lactation.

## Data Availability Statement

The datasets presented in this study can be found in online repositories. The names of the repository/repositories and accession number(s) can be found below: NCBI BioProject, Accession no: PRJNA699733 https://dataview.ncbi.nlm.nih.gov/object/PRJNA699733?reviewer=er592n5eopqpmo65ljj7983hh2 and NCBI SRR13638390.

## Ethics Statement

The animal study was reviewed and approved by the Animal Care and Use Committee of the Northwest A&F University.

## Author Contributions

YZ designed the project, performed the experiments, and wrote the manuscript. GLN, JDL, FJC and XPA helped with performing the experiments. BYC and QW applied for the funding for this study. All authors contributed to the article and approved the submitted version.

## Conflict of Interest

The authors declare that the research was conducted in the absence of any commercial or financial relationships that could be construed as a potential conflict of interest.

## Footnotes

1 http://www.ensembl.org/

2 http://geneontology.org/page/go-database

3 http://www.kegg.jp/kegg/ko.html
