# Supplementary figures and images for: EGF-Induced miR-223 Modulates Goat Mammary Epithelial Cell Apoptosis and Inflammation via ISG15
Source: Front Cell Dev Biol. 2021 Jun 30;9:660933. doi: 10.3389/fcell.2021.660933 (PMC8277964; doi:10.3389/fcell.2021.660933)

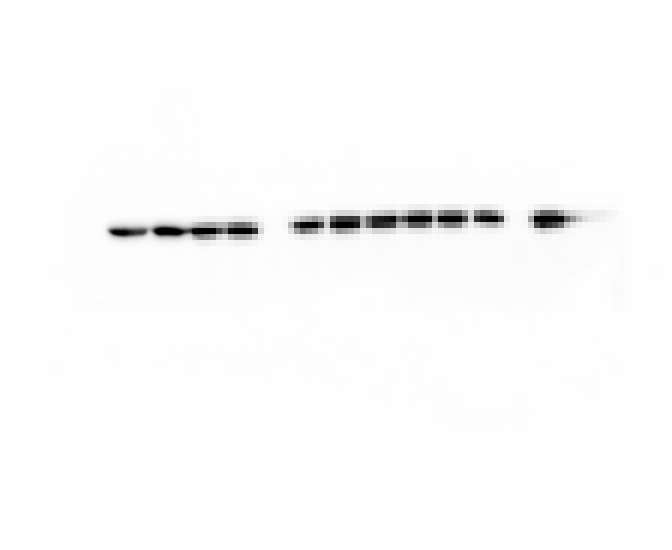

Supplement: Supplementary file 1 [file Data_Sheet_1.ZIP › 660933/western blots/Figure 3/3d b-actin Figure 3D.tif]

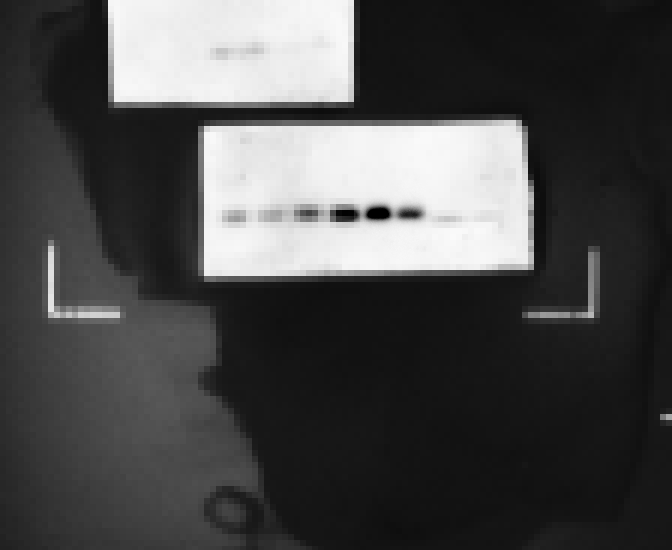

Supplement: Supplementary file 1 [file Data_Sheet_1.ZIP › 660933/western blots/Figure 3/3d ISG15 Figure 3D.tif]

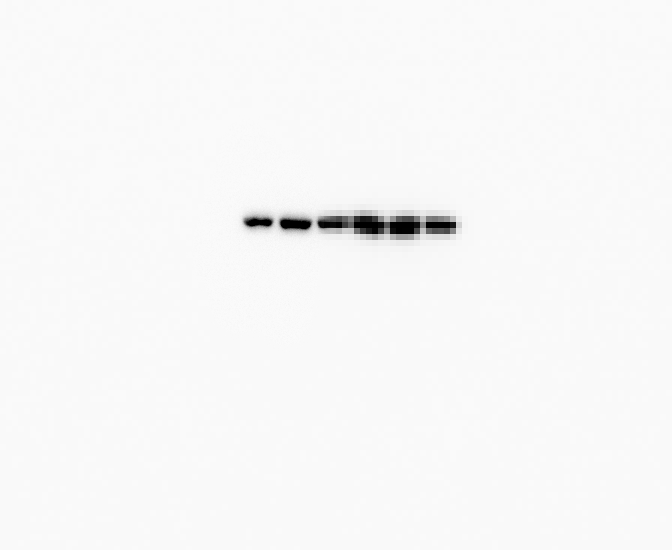

Supplement: Supplementary file 1 [file Data_Sheet_1.ZIP › 660933/western blots/Figure 3/3f b-actin left Figure 3F.tif]

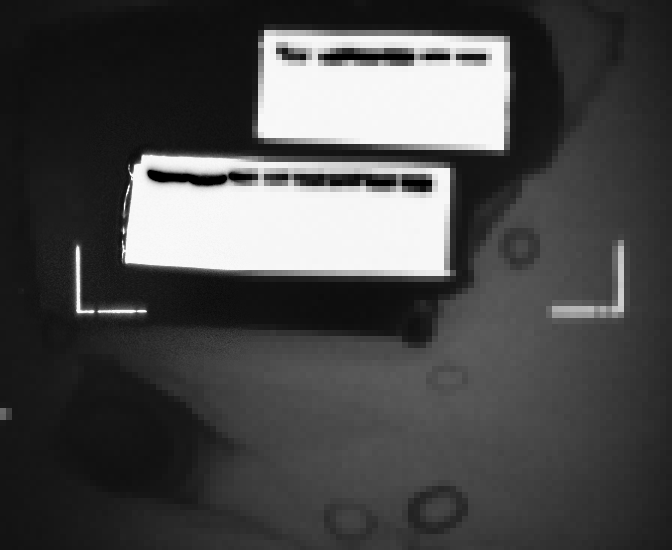

Supplement: Supplementary file 1 [file Data_Sheet_1.ZIP › 660933/western blots/Figure 3/3f b-actin right Figure 3F.tif]

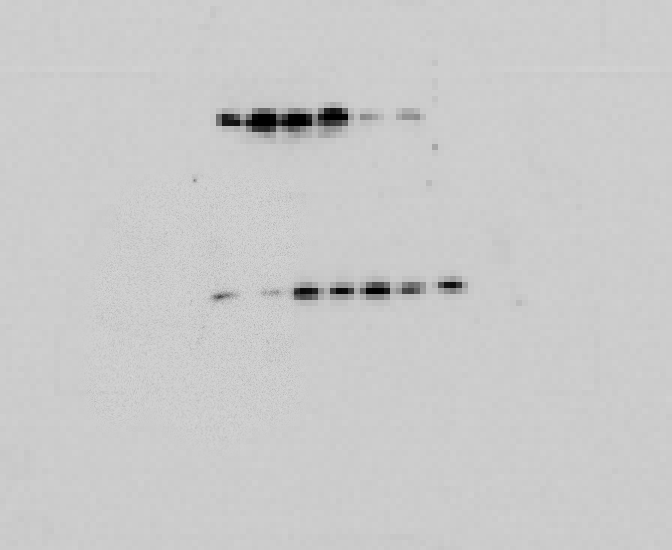

Supplement: Supplementary file 1 [file Data_Sheet_1.ZIP › 660933/western blots/Figure 3/3f ISG15 left Figure 3F.tif]

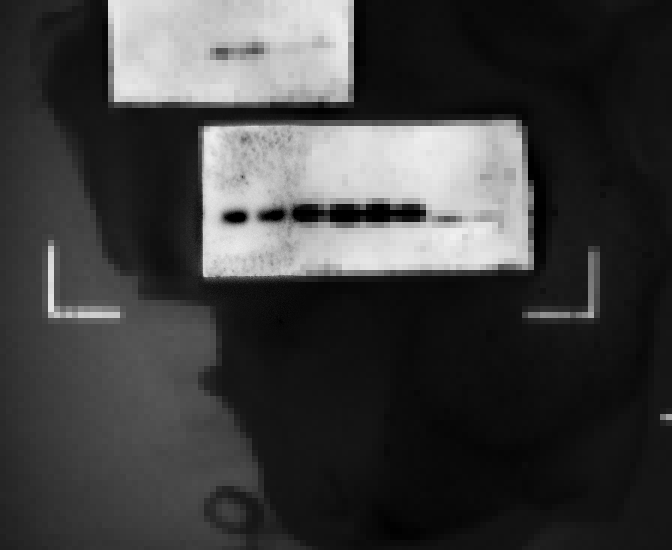

Supplement: Supplementary file 1 [file Data_Sheet_1.ZIP › 660933/western blots/Figure 3/3f ISG15 right Figure 3F.tif]

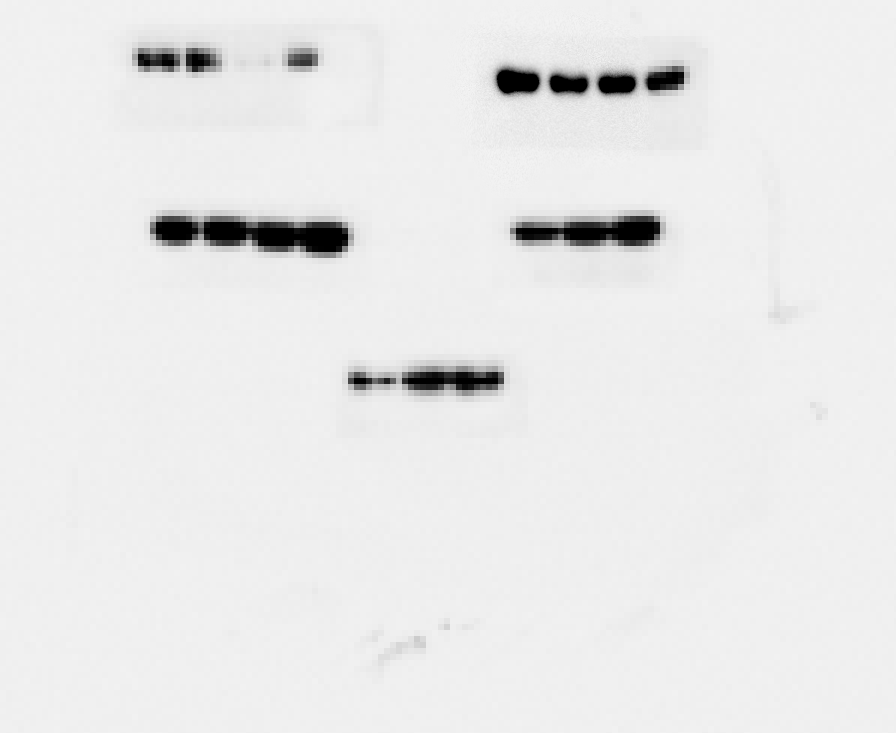

Supplement: Supplementary file 1 [file Data_Sheet_1.ZIP › 660933/western blots/Figure 3/3h b-actin lower Figure 3H.tif]

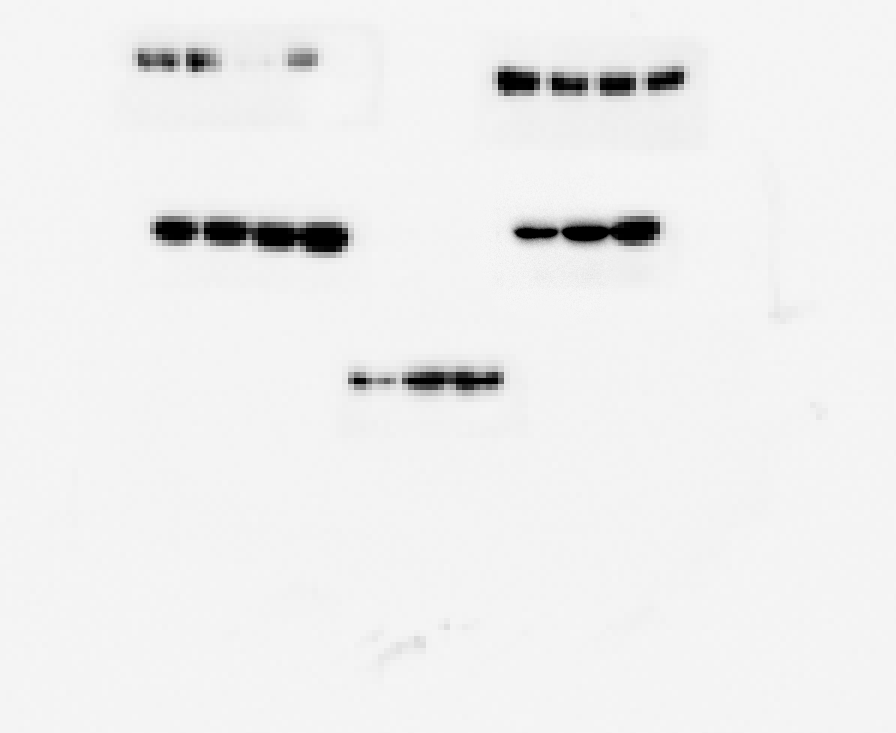

Supplement: Supplementary file 1 [file Data_Sheet_1.ZIP › 660933/western blots/Figure 3/3h b-actin upper Figure 3H.tif]

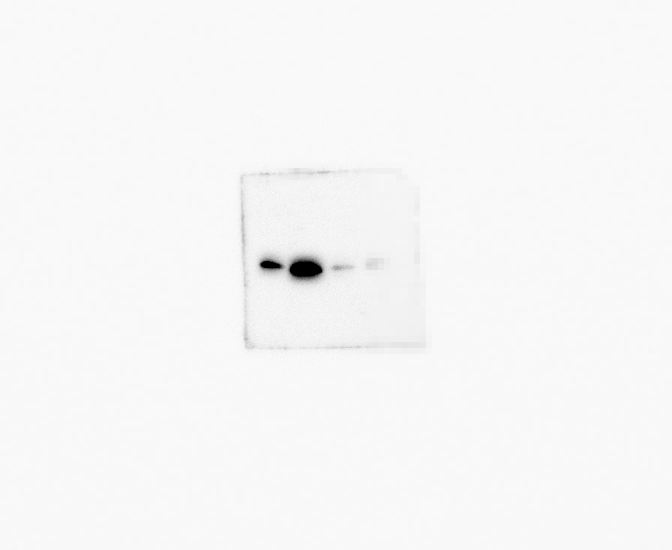

Supplement: Supplementary file 1 [file Data_Sheet_1.ZIP › 660933/western blots/Figure 3/3h ISG15 lower Figure 3H.tif]

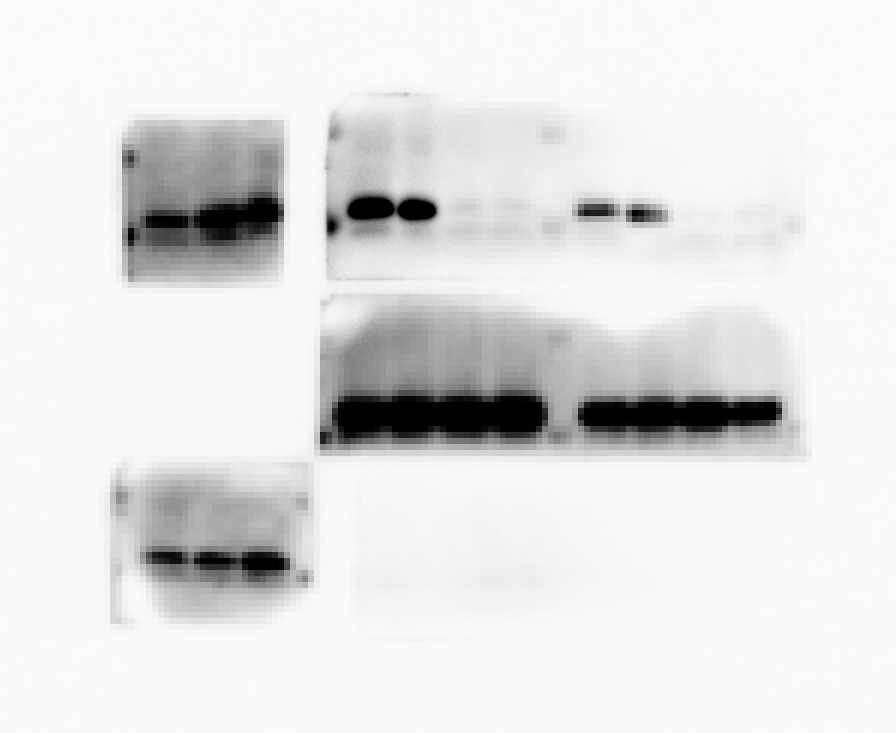

Supplement: Supplementary file 1 [file Data_Sheet_1.ZIP › 660933/western blots/Figure 3/3h ISG15 upper Figure 3H.tif]

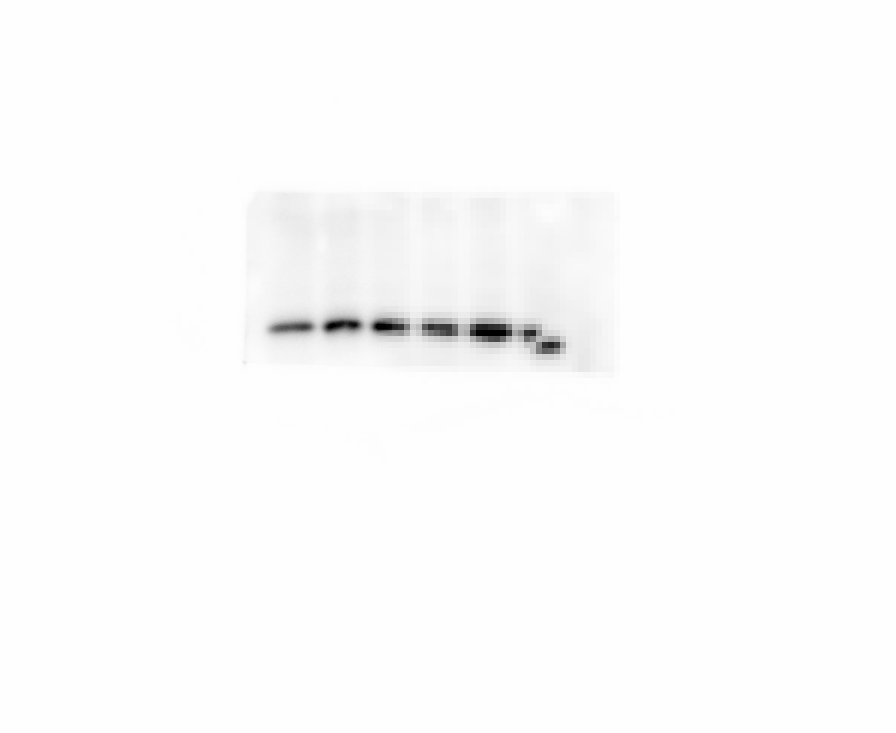

Supplement: Supplementary file 1 [file Data_Sheet_1.ZIP › 660933/western blots/Figure 3/3k Bax 1 Figure 3K.tif]

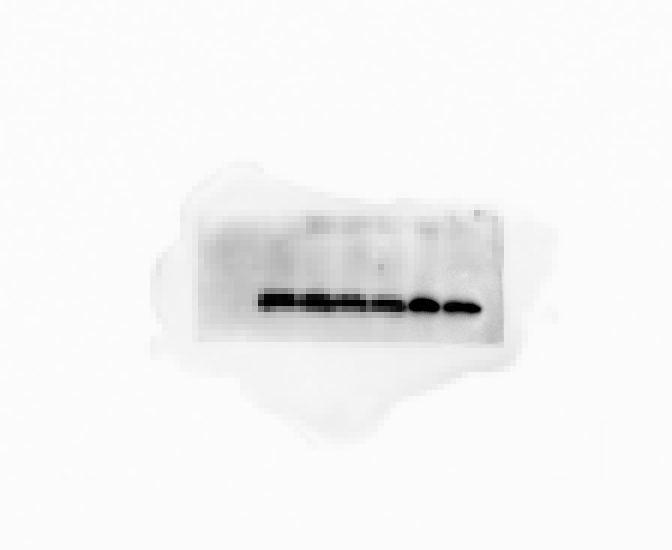

Supplement: Supplementary file 1 [file Data_Sheet_1.ZIP › 660933/western blots/Figure 3/3k Bax 2 Figure 3K.tif]

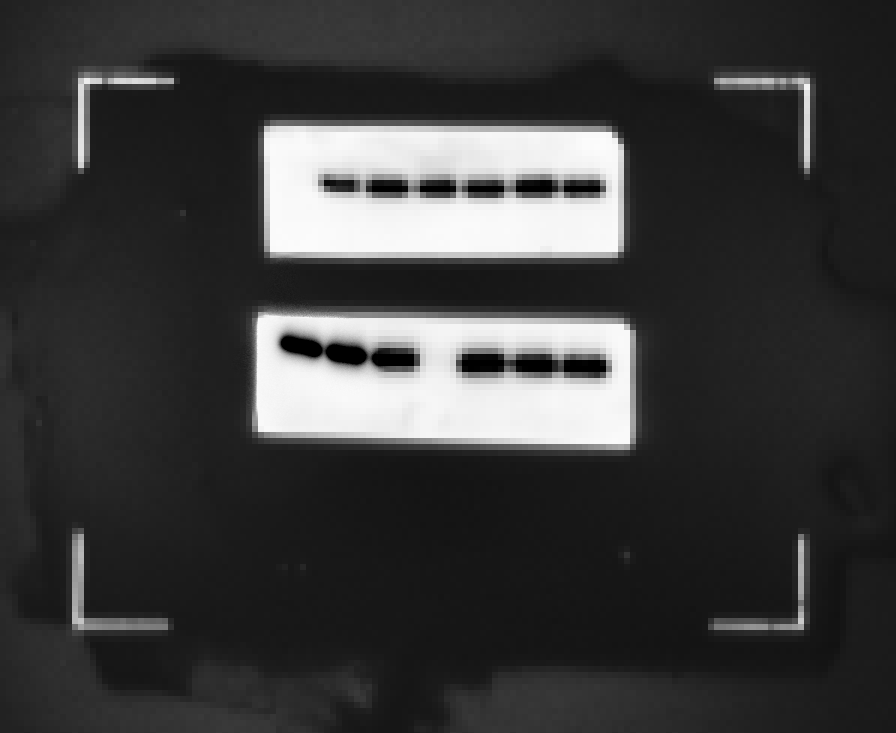

Supplement: Supplementary file 1 [file Data_Sheet_1.ZIP › 660933/western blots/Figure 3/3k Bax 3 Figure 3K.tif]

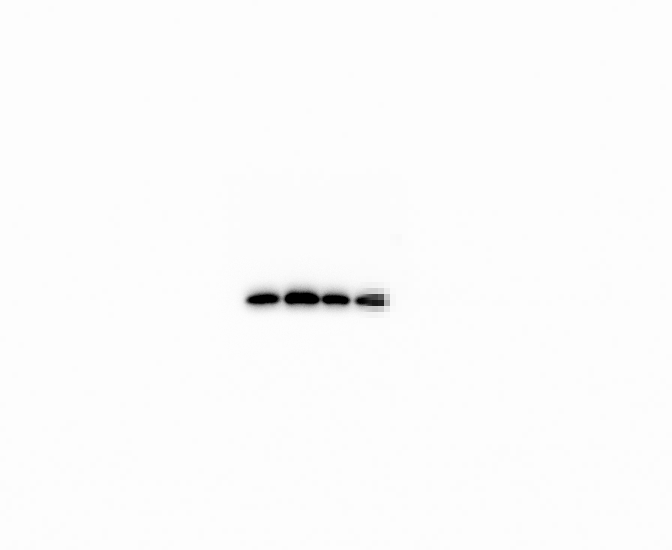

Supplement: Supplementary file 1 [file Data_Sheet_1.ZIP › 660933/western blots/Figure 3/3k Bax 4 Figure 3K.tif]

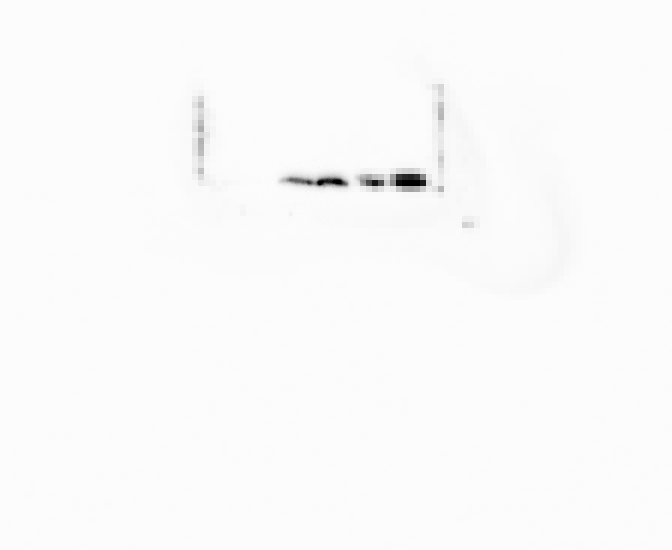

Supplement: Supplementary file 1 [file Data_Sheet_1.ZIP › 660933/western blots/Figure 3/3k Bcl-2 1 Figure 3K.tif]

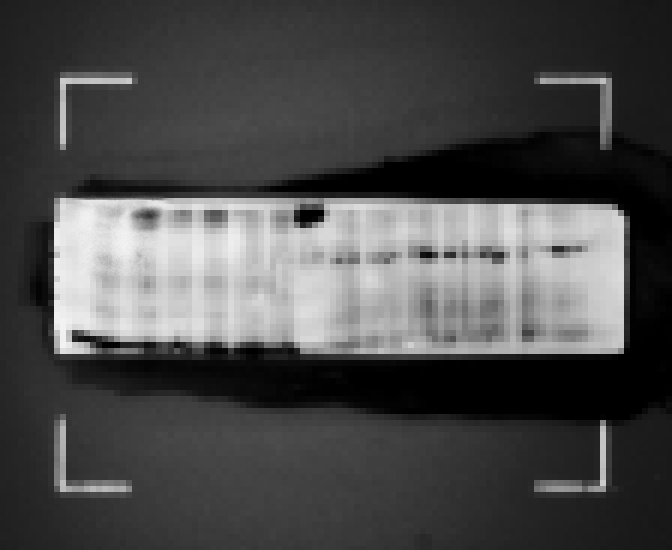

Supplement: Supplementary file 1 [file Data_Sheet_1.ZIP › 660933/western blots/Figure 3/3k Bcl-2 2 Figure 3K.tif]

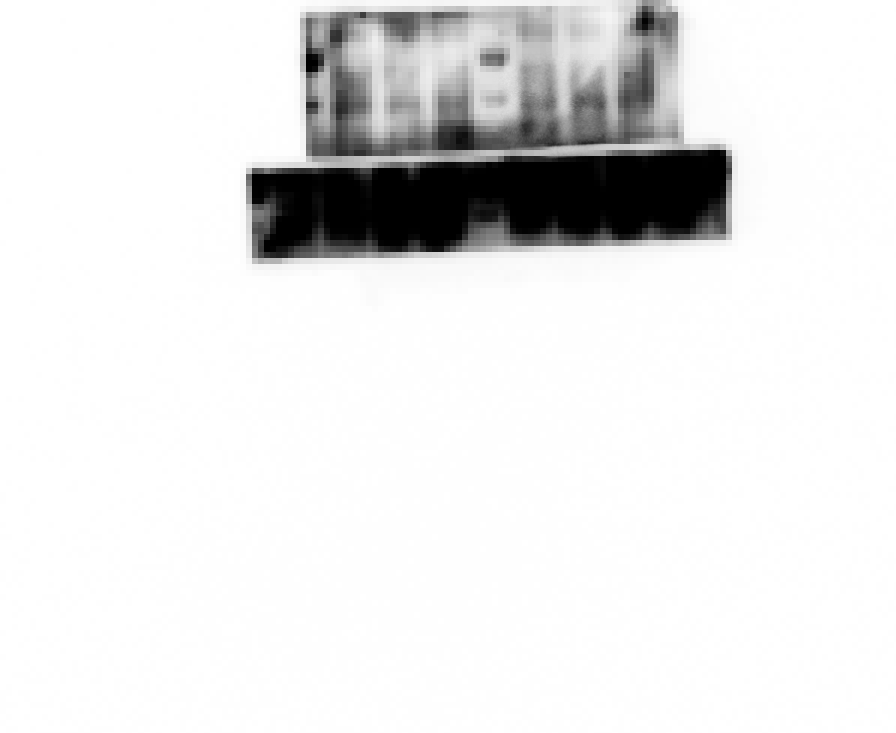

Supplement: Supplementary file 1 [file Data_Sheet_1.ZIP › 660933/western blots/Figure 3/3k Bcl-2 3 Figure 3K.tif]

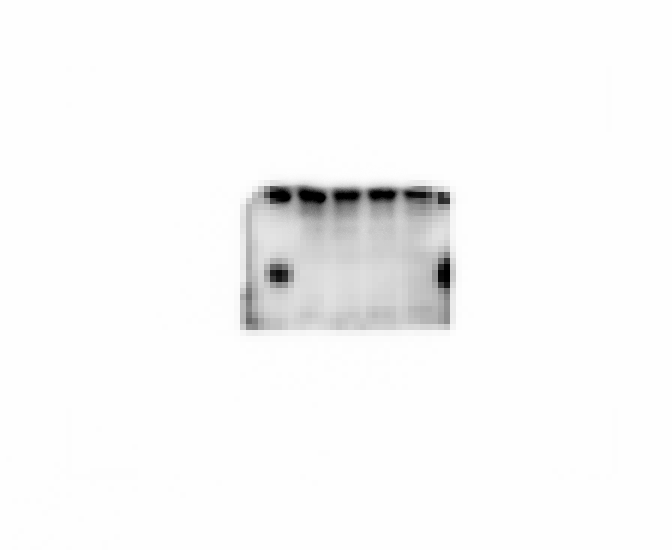

Supplement: Supplementary file 1 [file Data_Sheet_1.ZIP › 660933/western blots/Figure 3/3k Bcl-2 4 Figure 3K.tif]

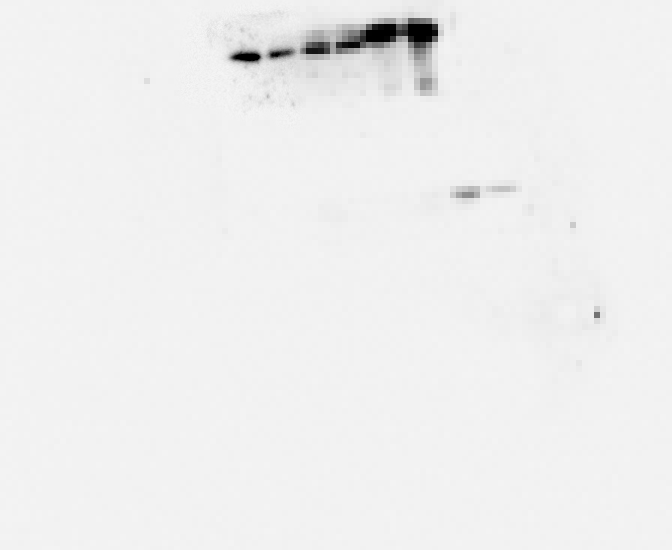

Supplement: Supplementary file 1 [file Data_Sheet_1.ZIP › 660933/western blots/Figure 3/3k Caspase3 1 Figure 3K.tif]

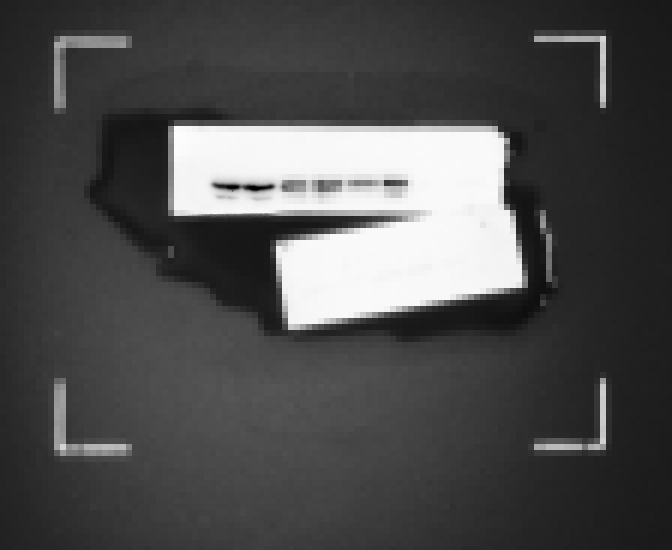

Supplement: Supplementary file 1 [file Data_Sheet_1.ZIP › 660933/western blots/Figure 3/3k Caspase3 2 Figure 3K.tif]

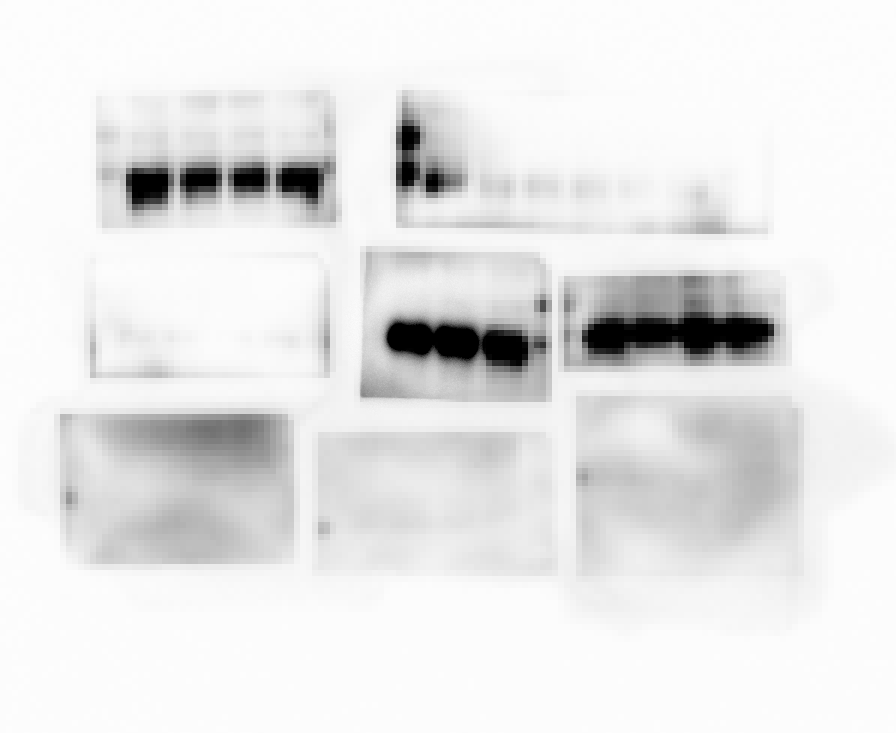

Supplement: Supplementary file 1 [file Data_Sheet_1.ZIP › 660933/western blots/Figure 3/3k Caspase3 3 Figure 3K.tif]

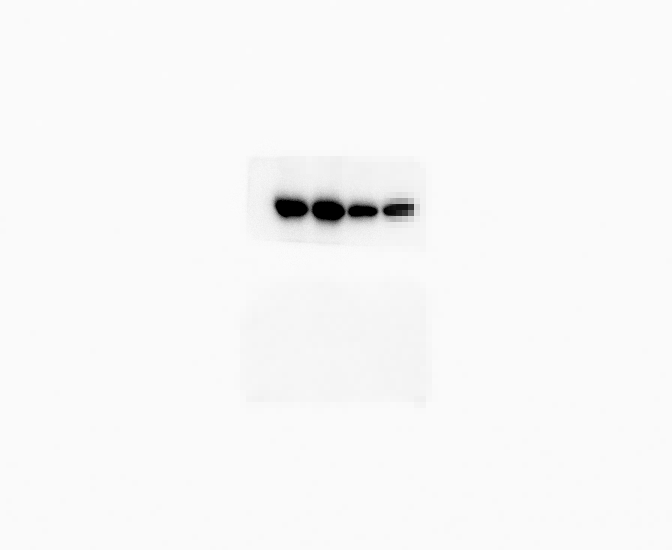

Supplement: Supplementary file 1 [file Data_Sheet_1.ZIP › 660933/western blots/Figure 3/3k Caspase3 4 Figure 3K.tif]

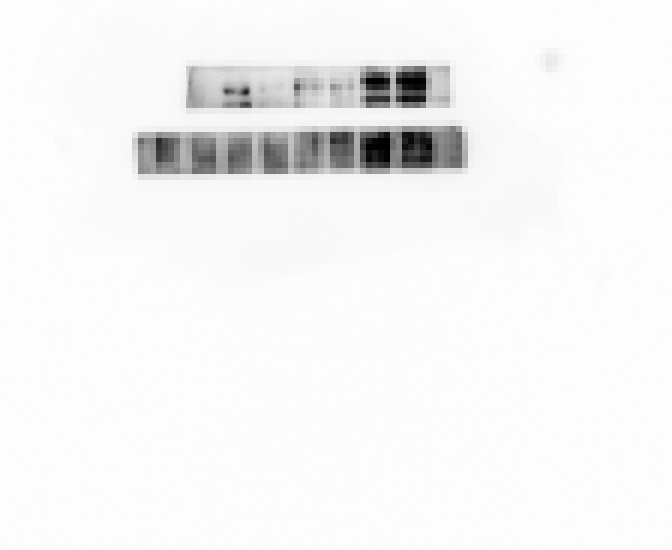

Supplement: Supplementary file 1 [file Data_Sheet_1.ZIP › 660933/western blots/Figure 3/3k p53 1 Figure 3K.tif]

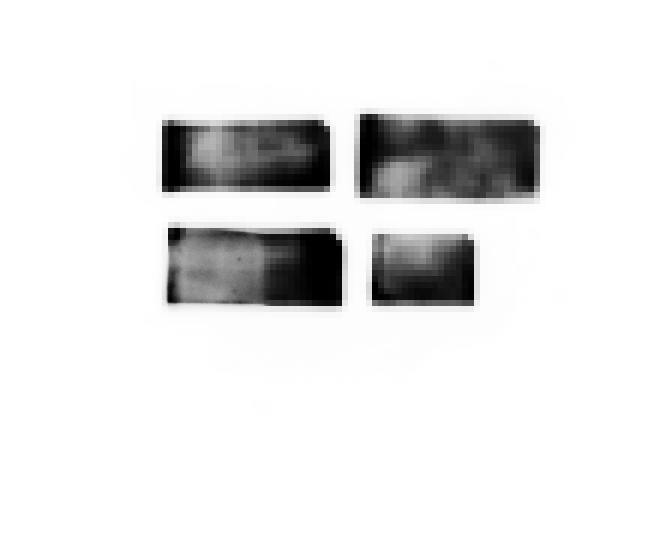

Supplement: Supplementary file 1 [file Data_Sheet_1.ZIP › 660933/western blots/Figure 3/3k p53 2 Figure 3K.tif]

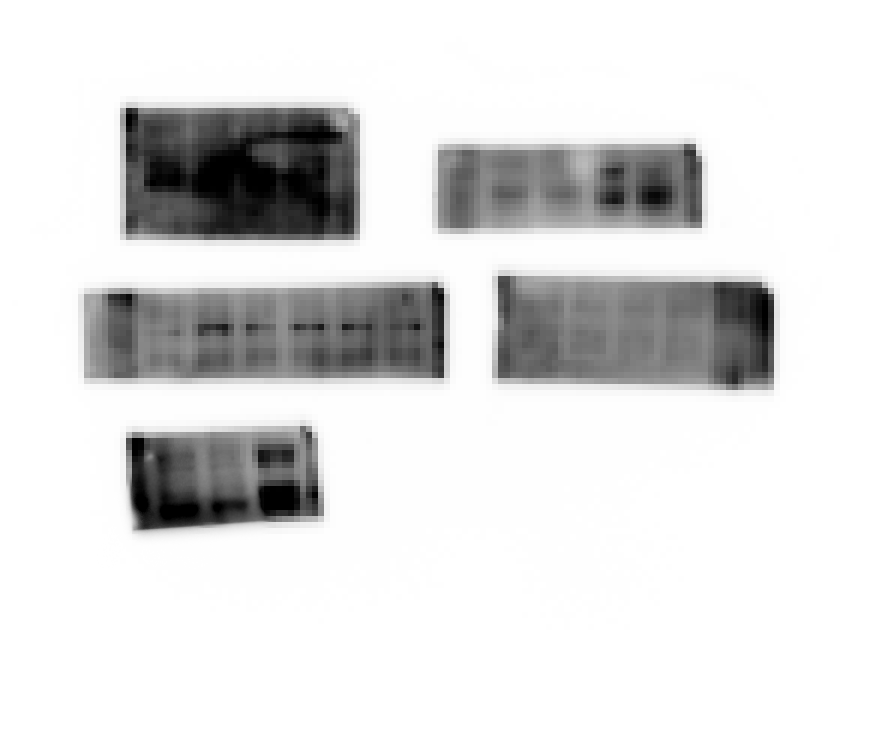

Supplement: Supplementary file 1 [file Data_Sheet_1.ZIP › 660933/western blots/Figure 3/3k p53 3 Figure 3K.tif]

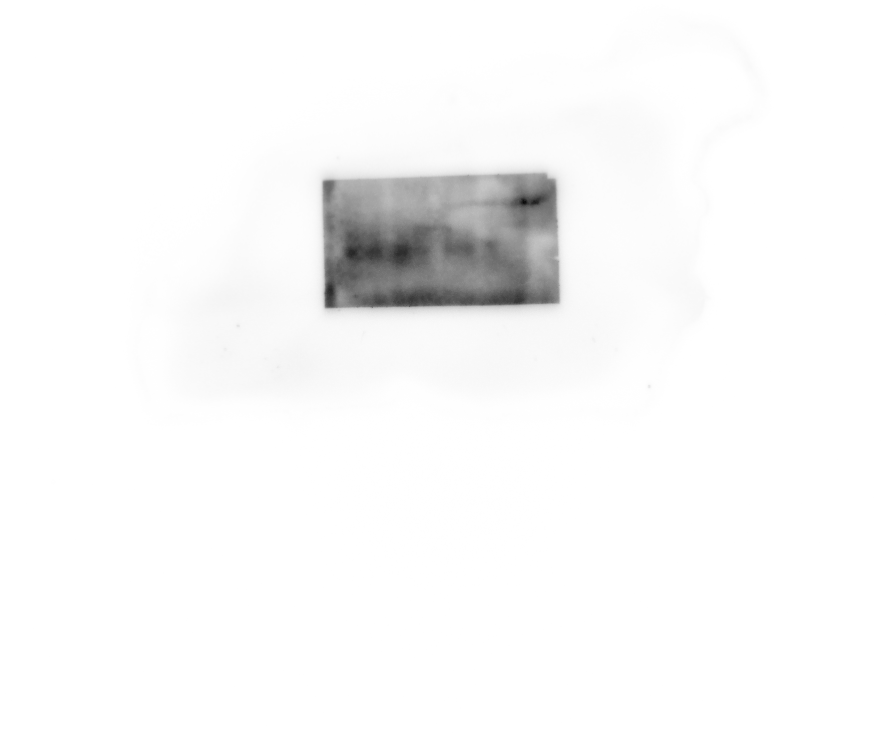

Supplement: Supplementary file 1 [file Data_Sheet_1.ZIP › 660933/western blots/Figure 3/3k p53 4 Figure 3K.tif]

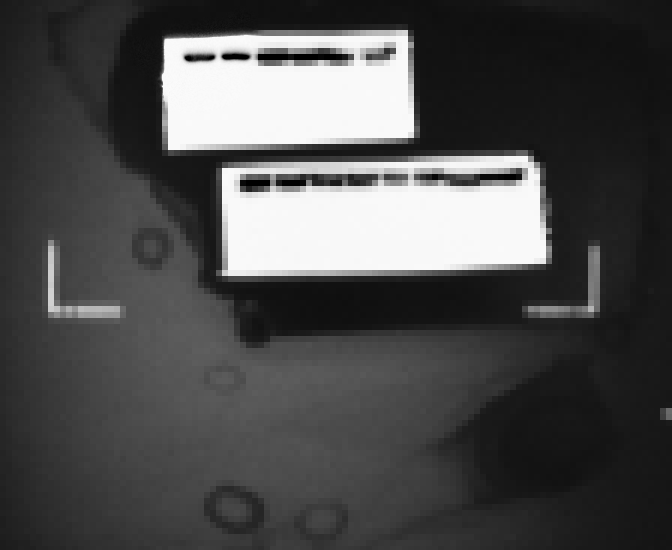

Supplement: Supplementary file 1 [file Data_Sheet_1.ZIP › 660933/western blots/Figure 4/4 b-actin 1 Figure 4.tif]

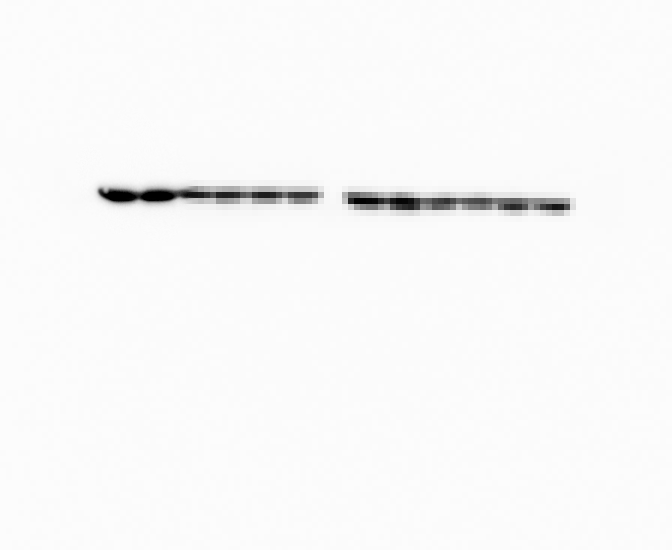

Supplement: Supplementary file 1 [file Data_Sheet_1.ZIP › 660933/western blots/Figure 4/4 b-actin 2 Figure 4.tif]

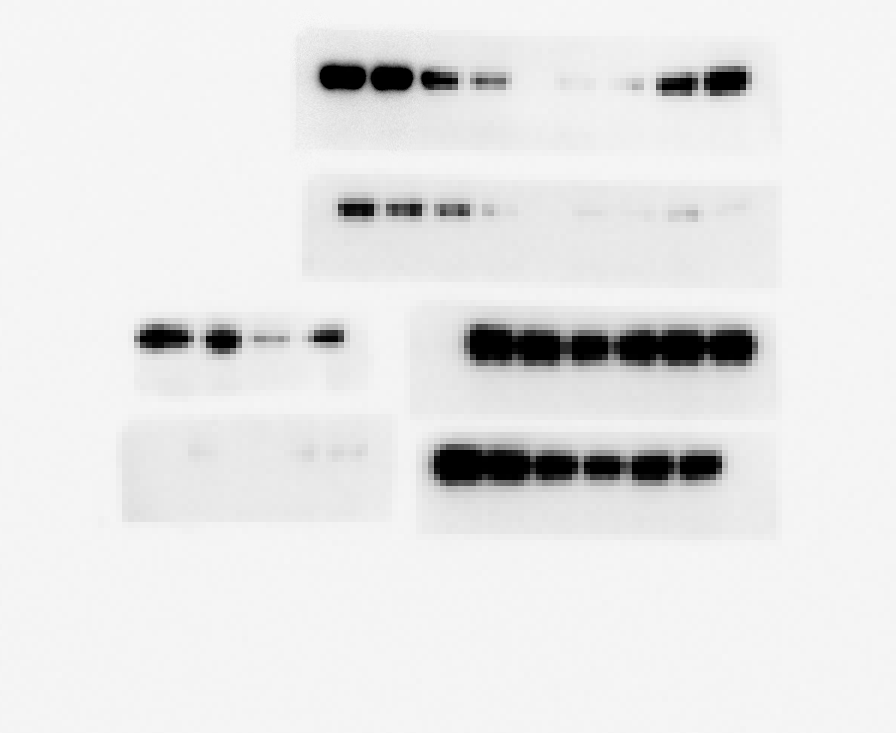

Supplement: Supplementary file 1 [file Data_Sheet_1.ZIP › 660933/western blots/Figure 4/4 b-actin 3 Figure 4.tif]

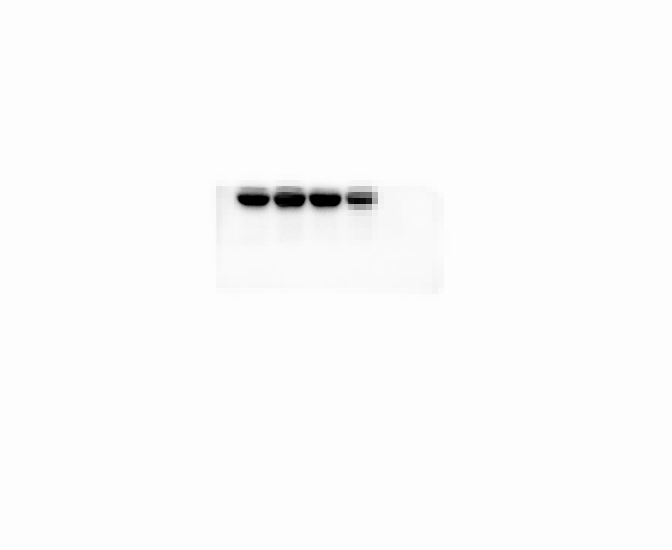

Supplement: Supplementary file 1 [file Data_Sheet_1.ZIP › 660933/western blots/Figure 4/4 b-actin 4 Figure 4.tif]

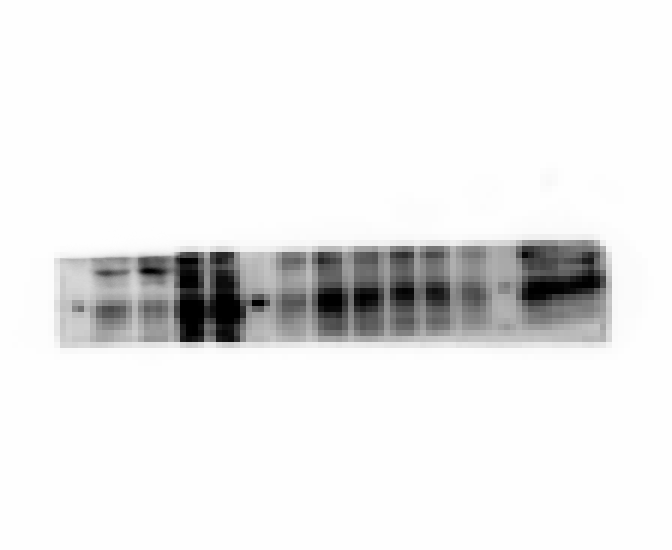

Supplement: Supplementary file 1 [file Data_Sheet_1.ZIP › 660933/western blots/Figure 4/4 FOXC1 1 Figure 4.tif]

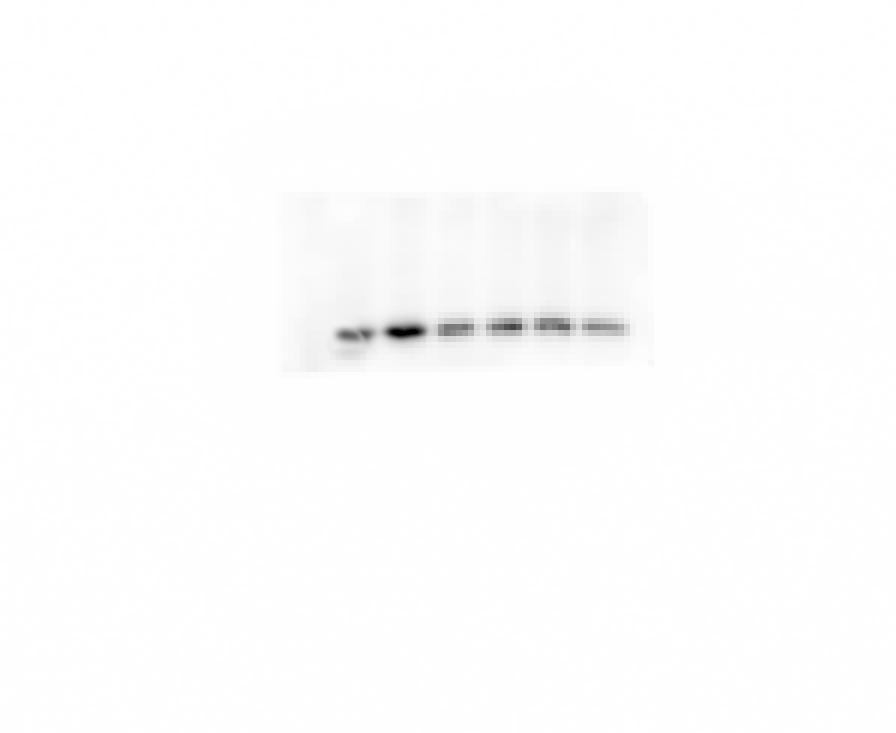

Supplement: Supplementary file 1 [file Data_Sheet_1.ZIP › 660933/western blots/Figure 4/4 FOXC1 2 Figure 4.tif]

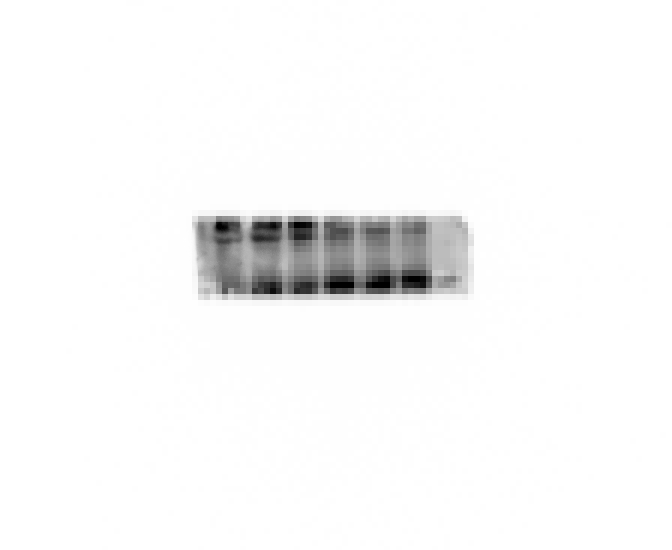

Supplement: Supplementary file 1 [file Data_Sheet_1.ZIP › 660933/western blots/Figure 4/4 FOXC1 3 Figure 4.tif]

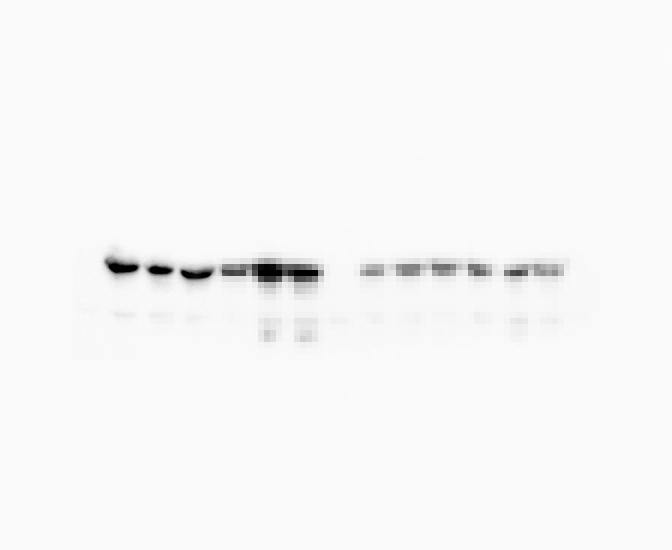

Supplement: Supplementary file 1 [file Data_Sheet_1.ZIP › 660933/western blots/Figure 4/4 FOXC1 4 Figure 4.tif]

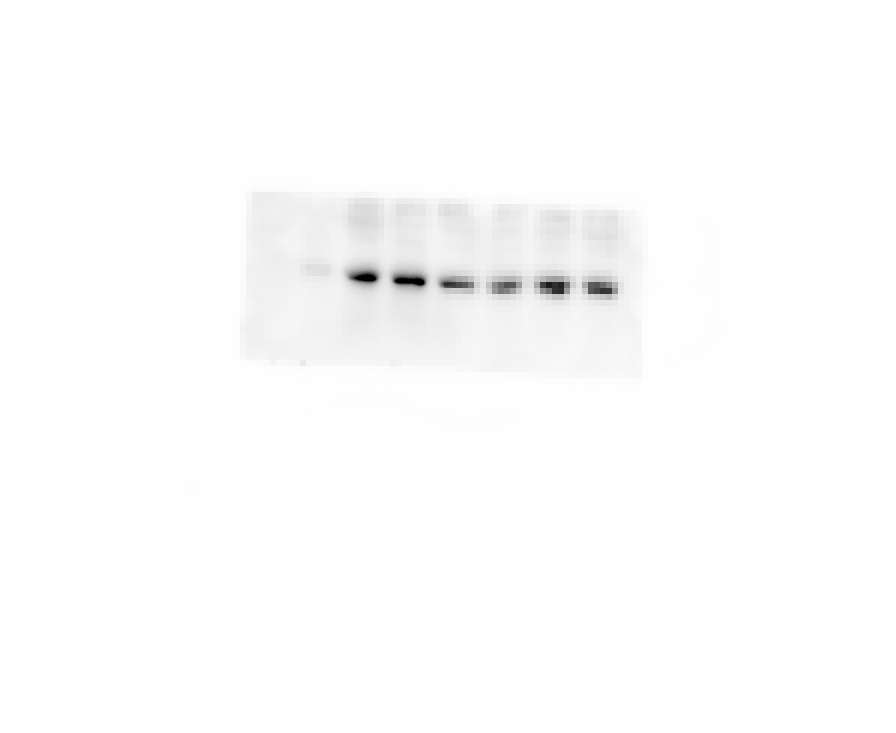

Supplement: Supplementary file 1 [file Data_Sheet_1.ZIP › 660933/western blots/Figure 4/4 HOXA10 1 Figure 4.tif]

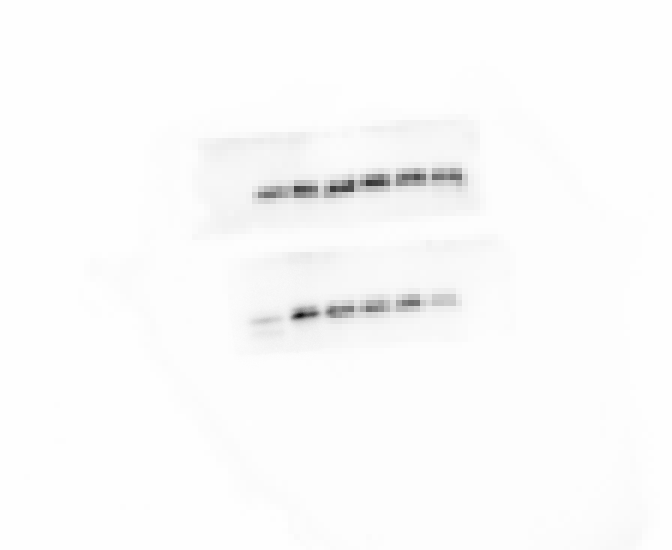

Supplement: Supplementary file 1 [file Data_Sheet_1.ZIP › 660933/western blots/Figure 4/4 HOXA10 2 Figure 4.tif]

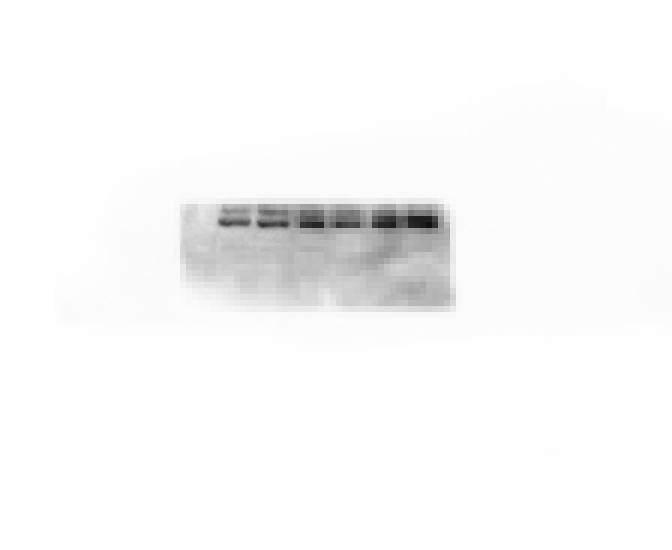

Supplement: Supplementary file 1 [file Data_Sheet_1.ZIP › 660933/western blots/Figure 4/4 HOXA10 3 Figure 4.tif]

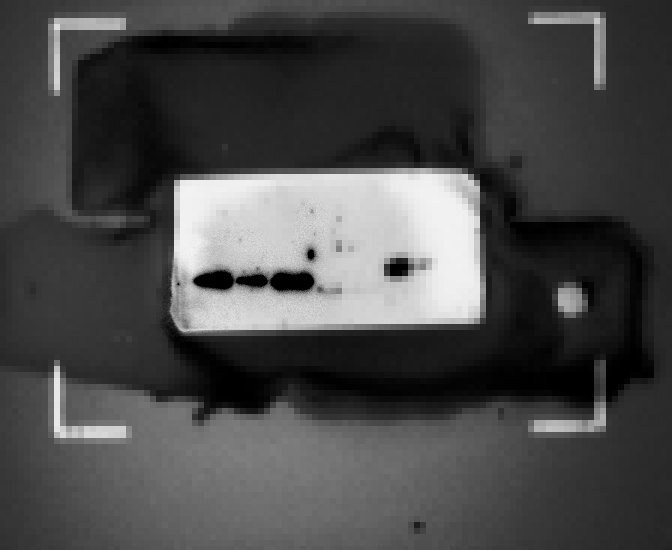

Supplement: Supplementary file 1 [file Data_Sheet_1.ZIP › 660933/western blots/Figure 4/4 HOXA10 4 Figure 4.tif]

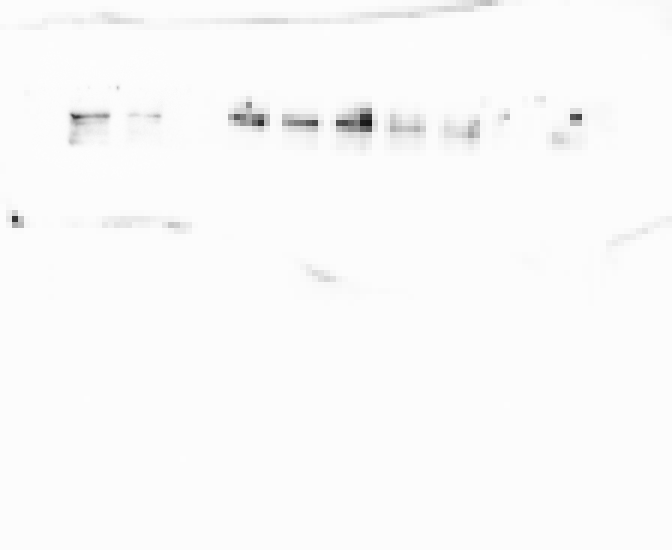

Supplement: Supplementary file 1 [file Data_Sheet_1.ZIP › 660933/western blots/Figure 4/4 p-STAT3 1 Figure 4.tif]

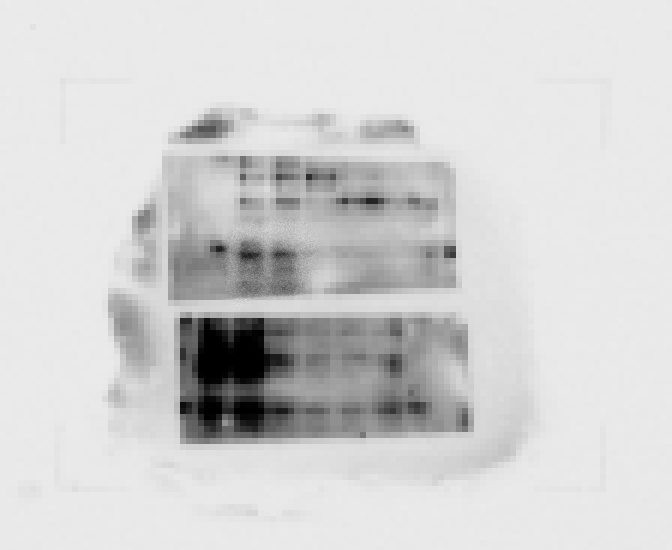

Supplement: Supplementary file 1 [file Data_Sheet_1.ZIP › 660933/western blots/Figure 4/4 p-STAT3 2 Figure 4.tif]

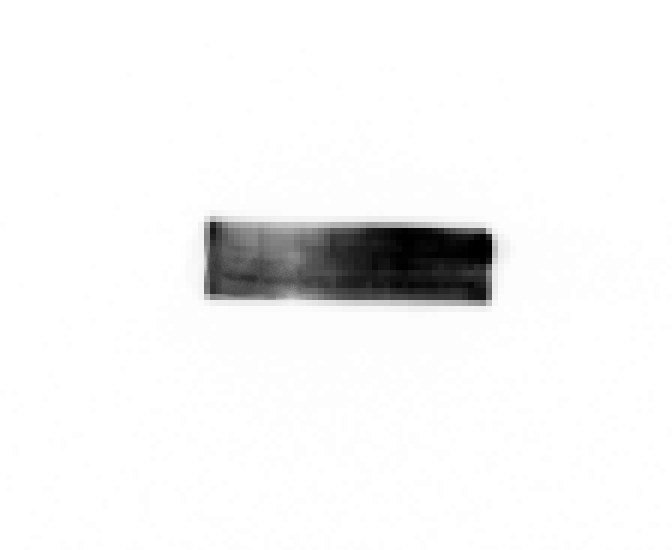

Supplement: Supplementary file 1 [file Data_Sheet_1.ZIP › 660933/western blots/Figure 4/4 p-STAT3 3 Figure 4.tif]

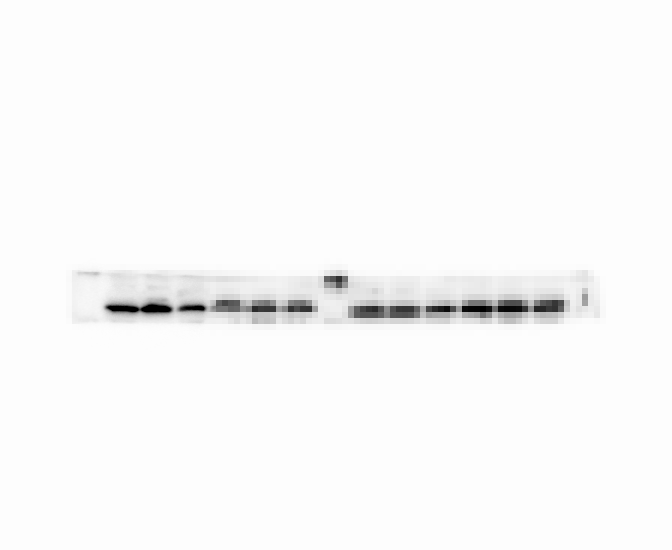

Supplement: Supplementary file 1 [file Data_Sheet_1.ZIP › 660933/western blots/Figure 4/4 p-STAT3 4 Figure 4.tif]

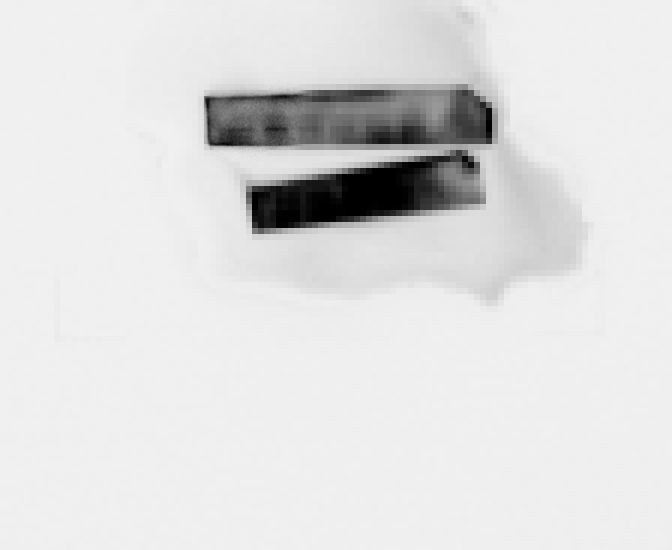

Supplement: Supplementary file 1 [file Data_Sheet_1.ZIP › 660933/western blots/Figure 4/4 PR 1 Figure 4.tif]

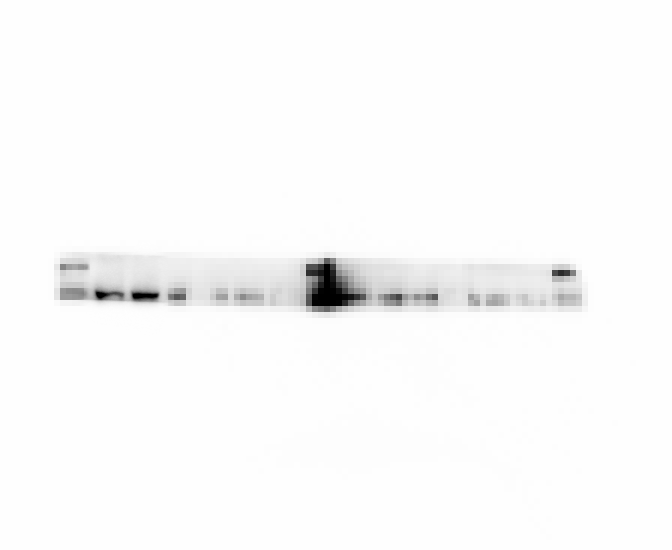

Supplement: Supplementary file 1 [file Data_Sheet_1.ZIP › 660933/western blots/Figure 4/4 PR 2 Figure 4.tif]

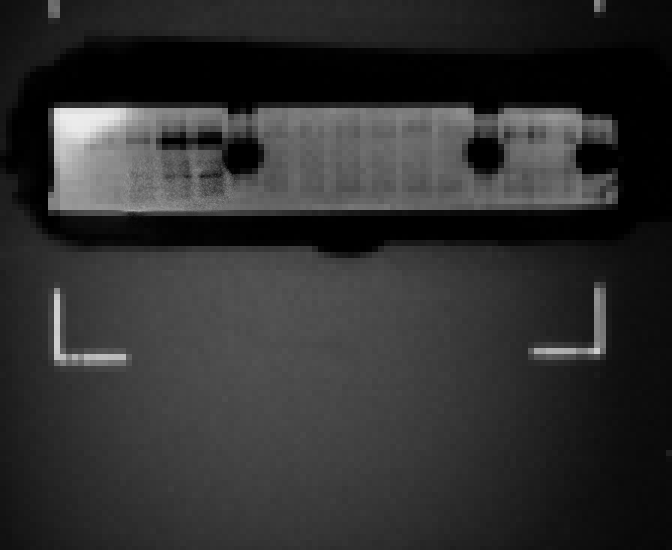

Supplement: Supplementary file 1 [file Data_Sheet_1.ZIP › 660933/western blots/Figure 4/4 PR 3 Figure 4.tif]

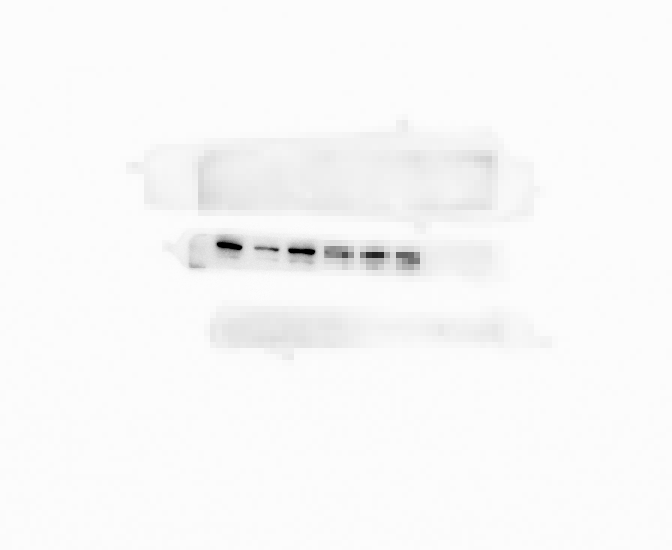

Supplement: Supplementary file 1 [file Data_Sheet_1.ZIP › 660933/western blots/Figure 4/4 PR 4 Figure 4.tif]

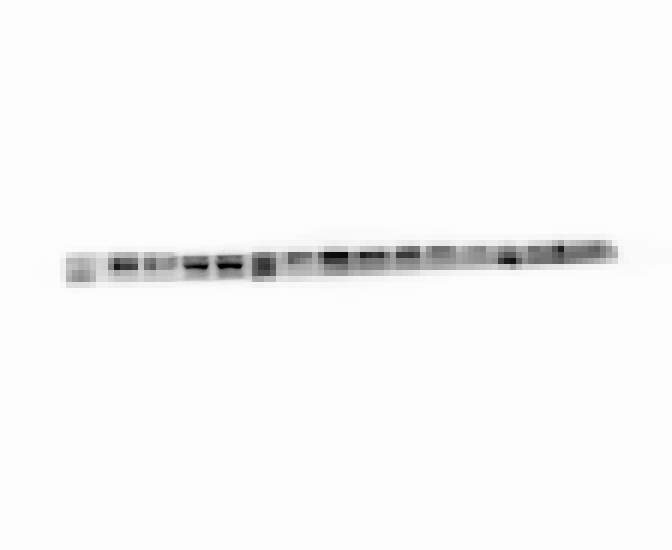

Supplement: Supplementary file 1 [file Data_Sheet_1.ZIP › 660933/western blots/Figure 4/4 STAT3 1 Figure 4.tif]

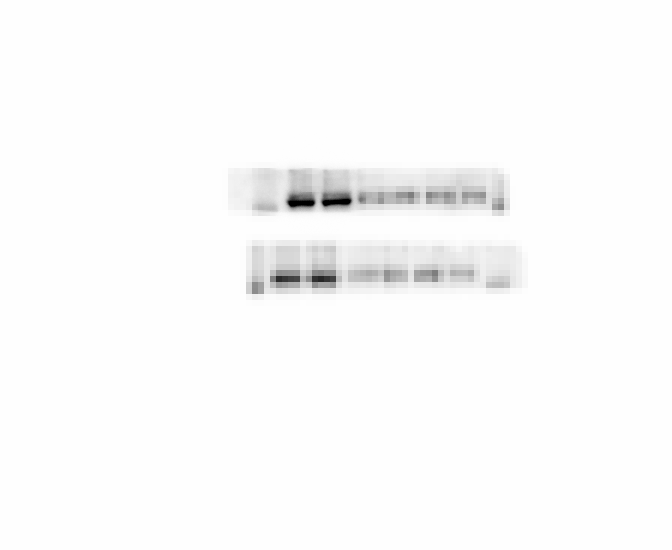

Supplement: Supplementary file 1 [file Data_Sheet_1.ZIP › 660933/western blots/Figure 4/4 STAT3 2 Figure 4.tif]

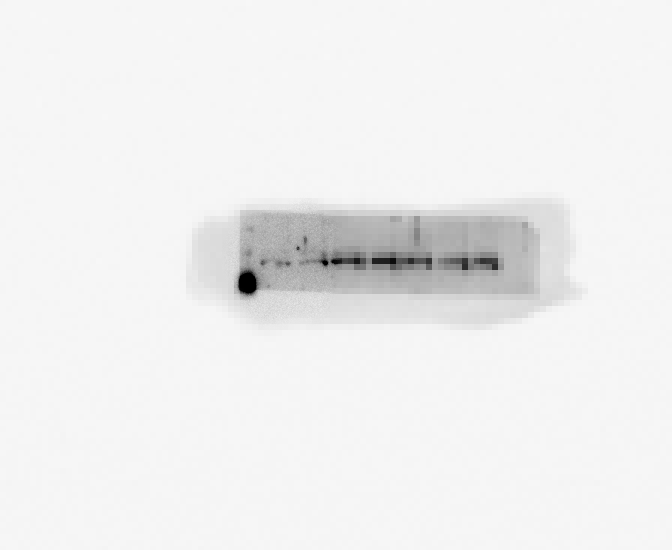

Supplement: Supplementary file 1 [file Data_Sheet_1.ZIP › 660933/western blots/Figure 4/4 STAT3 3 Figure 4.tif]

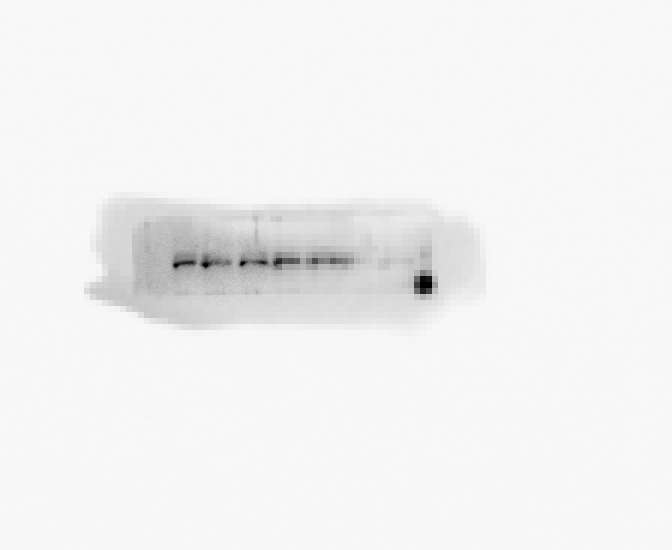

Supplement: Supplementary file 1 [file Data_Sheet_1.ZIP › 660933/western blots/Figure 4/4 STAT3 4 Figure 4.tif]

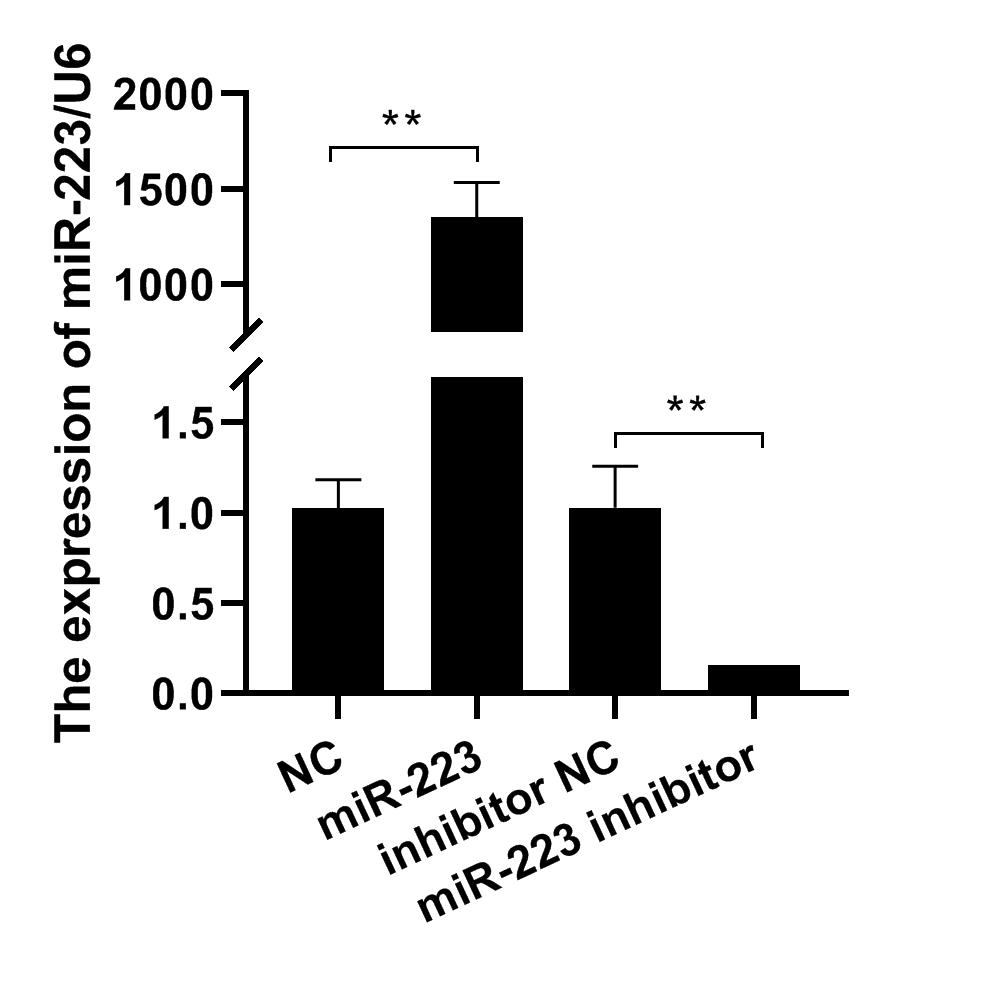

Supplement: Supplementary file 2 [file Image_1.JPEG]
